# Pathological changes induced by Alzheimer’s brain inoculation in amyloid-beta plaque-bearing mice

**DOI:** 10.1186/s40478-022-01410-y

**Published:** 2022-08-16

**Authors:** Suzanne Lam, Anne-Sophie Hérard, Susana Boluda, Fanny Petit, Sabiha Eddarkaoui, Karine Cambon, The Brainbank Neuro-CEB Neuropathology Network, Franck Letournel, Marie-Laure Martin-Négrier, Maxime Faisant, Catherine Godfraind, Jean Boutonnat, Claude-Alain Maurage, Vincent Deramecourt, Mathilde Duchesne, David Meyronet, Tanguy Fenouil, André Mauès de Paula, Valérie Rigau, Fanny Vandenbos-Burel, Danielle Seilhean, Charles Duyckaerts, Susana Boluda, Isabelle Plu, Dan Christian Chiforeanu, Annie Laquerrière, Florent Marguet, Béatrice Lannes, Benoît Lhermitte, Jean-Luc Picq, Luc Buée, Charles Duyckaerts, Stéphane Haïk, Marc Dhenain

**Affiliations:** 1 CEA, CNRS, Laboratoire des Maladies Neurodégénératives, MIRCen, Université Paris-Saclay, 18 Route du Panorama, 92265 Fontenay-aux-Roses, France; 2 Direction de la Recherche Fondamentale (DRF), Institut François Jacob, MIRCen, Commissariat à l’Energie Atomique et aux Energies Alternatives (CEA), 18 Route du Panorama, 92265 Fontenay-aux-Roses, France; 3 ICM Institut du Cerveau et de la Moelle épinière, CNRS UMR7225, INSERM U1127, Sorbonne Université, Hôpital de la Pitié-Salpêtrière, Paris, France; 4 Brainbank NeuroCEB Neuropathology Network: Plate-Forme de Ressources Biologiques, Bâtiment Roger Baillet, Hôpital de la Pitié-Salpêtrière, 47-83 boulevard de l’Hôpital, 75651 Paris Cedex 13, France; 5 Inserm, CHU-Lille, Lille Neuroscience & Cognition, Alzheimer & Tauopathies, LabEx DISTALZ, Université de Lille, Rue Polonovski, 59045 Lille, France; 6 Laboratory of Cognitive Functioning and Dysfunctioning (DysCo), University Paris 8, 93526 Saint-Denis Cedex, France

**Keywords:** Alzheimer's disease, Amyloid-β, Memory, Microglia, Synapses, Tau, Transmission

## Abstract

Alzheimer's disease (AD) is characterized by intracerebral accumulations of extracellular amyloid-β (Aβ) plaques and intracellular tau pathology that spread in the brain. Three types of tau lesions occur in the form of neuropil threads, neurofibrillary tangles, and neuritic plaques i.e. tau aggregates within neurites surrounding Aβ deposits. The cascade of events linking these lesions and synaptic or memory impairments are still debated. Intracerebral infusion of human AD brain extracts in Aβ plaque-bearing mice that do not overexpress pathological tau proteins induces tau pathologies following heterotopic seeding of mouse tau protein. There is however little information regarding the downstream events including synaptic or cognitive repercussions of tau pathology induction in these models. In the present study, human AD brain extracts (AD_be_) and control-brain extracts (Ctrl_be_) were infused into the hippocampus of Aβ plaque-bearing APP_swe_/PS1_dE9_ mice. Memory, synaptic density, as well as Aβ plaque and tau aggregate loads, microgliosis, astrogliosis at the inoculation site and in connected regions (perirhinal/entorhinal cortex) were evaluated 4 and 8 months post-inoculation. AD_be_ inoculation produced the following effects: (i) memory deficit; (ii) increased Aβ plaque deposition in proximity to the inoculation site; (iii) tau pathology induction; (iv) appearance of neuropil threads and neurofibrillary tangles next to the inoculation site with a spreading to connected regions. Neuritic plaque pathology was detected in both AD_be_- and Ctrl_be_-inoculated animals but AD_be_ inoculation increased the severity close to and at distance of the inoculation site. (v) Finally, AD_be_ inoculation reduced synaptic density in the vicinity to the inoculation site and in connected regions as the perirhinal/entorhinal cortex. Synaptic impairments were correlated with increased severity of neuritic plaques but not to other tau lesions or Aβ lesions, suggesting that neuritic plaques are a culprit for synaptic loss. Synaptic density was also associated with microglial load.

**Graphical abstract:**

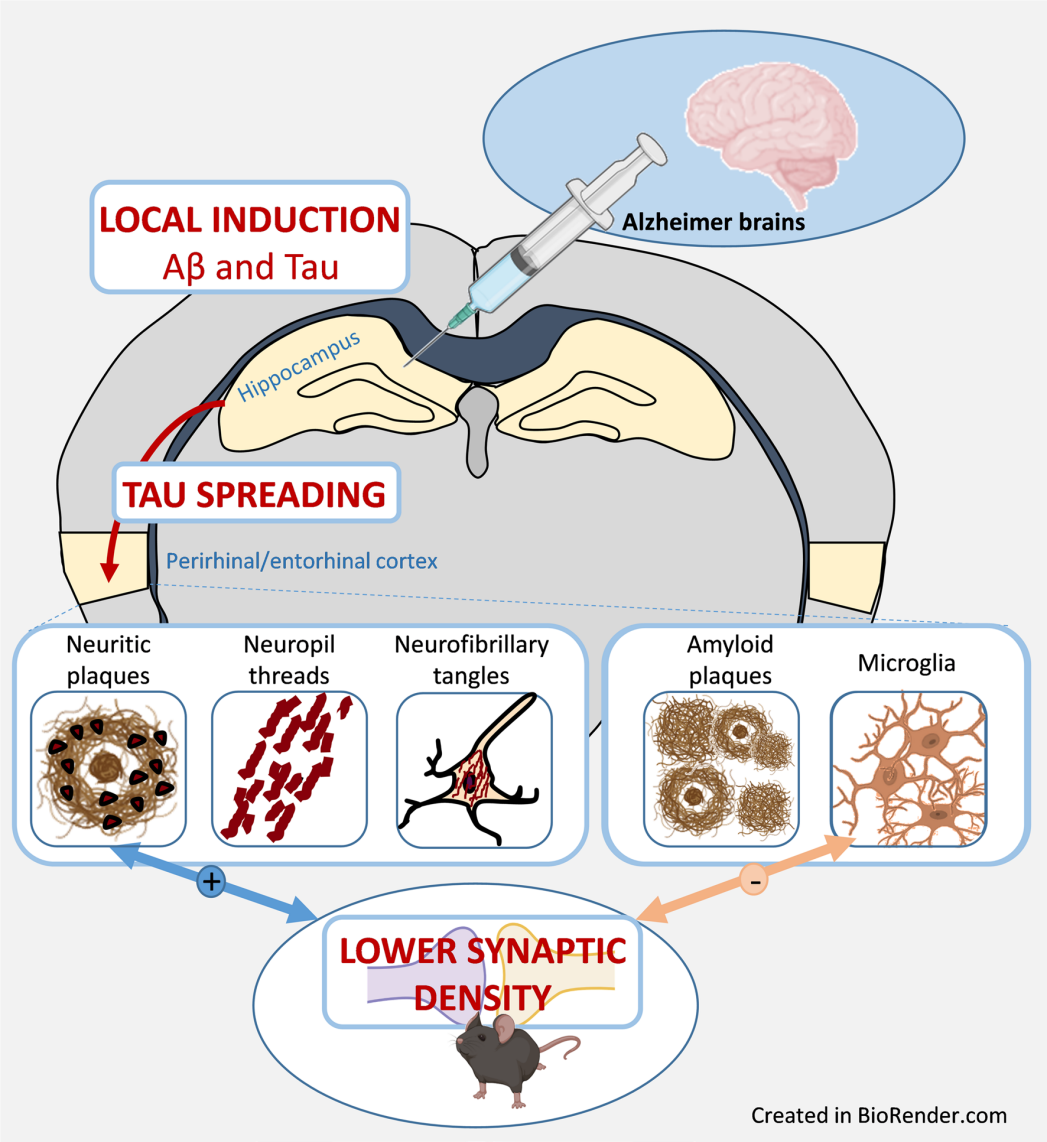

**Supplementary Information:**

The online version contains supplementary material available at 10.1186/s40478-022-01410-y.

## Introduction

Alzheimer’s disease (AD) is a clinical entity leading to cognitive deficits. AD core lesions include amyloid-β (Aβ) plaques and intracellular tau accumulations that spread in a highly stereotyped pattern through the brain [3]. In humans, during clinically established AD, memory impairment is correlated with neocortical tau pathology and with synaptic alterations but not so directly with Aβ plaque load [2, 18, 29]. Transgenic animals are widely used to investigate the pathophysiological mechanisms associated with AD. The association between synaptic deficits and Aβ pathology was reported in transgenic (Tg) mouse models overexpressing Aβ [25–27] while the association between synaptic deficits and tau pathology was outlined in Tg models overexpressing tau protein (with or without frontotemporal dementia mutations) [6, 12, 21, 27]. These results suggest a role of both Aβ and tau pathologies in the induction of synaptic deficits in rodents, but the relative participation of Aβ and tau lesions to synaptic impairments remains undefined and unquantified.

As AD is characterized by both Aβ and tau pathologies, it is necessary to investigate its pathophysiology in animal models presenting with both Aβ and tau lesions. Mouse models of amyloidosis were shown to display tau lesions mainly in neurites close to Aβ deposits [16, 19, 22]. Presumably due to the low level of tau pathology, these animals are not used as models of both Aβ and tau pathologies. Novel categories of models with both Aβ and tau pathologies were recently established by infusing human AD brain extracts (AD_be_) in the brain of Aβ plaque-bearing mice that do not overexpress pathological tau proteins [11, 30]. These models rely on the notion that tau proteins have prion‐like properties and can be experimentally transmitted. Once in the cell environment, they are incorporated by unaffected cells, and then amplified by seeding the aggregation of endogenous tau. In these models, tau seeds from AD_be_ induce the heterotopic seeding of mouse tau [1, 30]. These models have been mainly used to investigate the relationships between Aβ and tau pathologies [11, 15, 30]. The relationships between AD core lesions and downstream events as synaptic impairments have not been reported, and memory changes remain mostly undefined in these models [11, 30]. To address these questions, we infused AD_be_ as well as control brain extracts (Ctrl_be_) in the hippocampus of APP_swe_/PS1_dE9_ mice that have a high Aβ production, express endogenous murine tau protein isoforms and are not transgenic for any human tau. Infusion of AD_be_ increased Aβ load at the inoculation site as well as tau lesions that spread in connected areas notably the perirhinal/entorhinal cortex. AD_be_–inoculated animals displayed memory and synaptic impairments. Synaptic defects were correlated with neuritic tau lesions but not with other tau lesions or Aβ pathology. Neuroinflammation was additionally evaluated as a complementary measure. Unexpectidly, synaptic density and microglial load were correlated.

## Methods

### Human brain collection and characterization

Frozen brain samples (parietal cortex) from eight sporadic AD patients as well as two age-matched control individuals were collected from a brain donation program of the NeuroCEB and the CNR-prion brain banks (Additional file 1: Table S1). Consent forms were previously signed by either the patients themselves or their next of kin, in accordance with French bioethics laws. AD patients had a classical evolving form of the pathology, characterized by a disease duration of 5 to 8 years (n = 4) or a more rapidly evolving form characterized by a disease duration of 6 months to 3 years (n = 4). No case of hippocampal sclerosis was reported and all brain samples were PrPSc negative. Samples from AD brains were also negative for α-synuclein and TDP-43. All brain tissues were assessed by immunohistochemistry, as previously described in Lam et al. 2021 [14]. Briefly, 4-μm-thick paraffin sections were cut, deparaffinized in xylene, successively rehydrated in ethanol (100, 90, and 70%) and rinsed under running tap water for 10 min before immunohistological staining. They were then incubated in 99% formic acid for 5 min, quenched for endogenous peroxidase with 3% hydrogen peroxide and 20% methanol, and washed in water. Sections were blocked at room temperature for 30 min in 4% bovine serum albumin (BSA) in 0.05 M tris-buffered saline, with 0.05% Tween 20, pH 8 (TBS-Tween, Sigma). They were then incubated overnight at + 4 °C with the 6F3D anti-Aβ antibody (Dako, 1/200), polyclonal anti-tau antibody (Dako, 1/500), monoclonal anti-alpha-synuclein (LB509, Zymed, 1/250), polyclonal anti-TDP43 (Protein Tech Group, 1/1000) routinely used for Aβ, tau, alpha-synuclein and TDP43 detection, respectively. Sections were further incubated with a biotinylated secondary antibody for 25 min at room temperature, and the presence of the secondary antibody was revealed by a streptavidin–horseradish peroxidase conjugate using diaminobenzidine (Dako, Glostrup, Denmark). Slices were counterstained with Harris hematoxylin.

Brain samples were also evaluated by biochemistry. For tau protein extraction, brain homogenates were sonicated on ice for 5 min, centrifuged for 5 min at 3000 × g at + 4 °C, diluted in 20 mM Tris/2% SDS and sonicated on ice for 5 min. For Aβ, Iba1 and GFAP protein extractions, brain homogenates were sonicated (6 strokes, cycle 0.5, 30% amplitude) in a lysis buffer at a final concentration of 50 mM Tris–HCl pH 7.4, 150 mM NaCl, 1% Triton-X-100 supplemented with 1X protease inhibitors (Complete^™^ Mini, EDTA-free Protease Inhibitor Cocktail, Roche) and 1/100 diluted phosphatase inhibitors (Phosphatase Inhibitor Cocktail 2, Sigma-Aldrich). Samples were centrifuged at 20,000 × g for 20 min at + 4 °C and the supernatant was collected for further use. Extracted samples were stored at − 80 °C after evaluation of total protein concentration by a BCA assay (Pierce^™^). For tau characterization, samples were diluted to 1 μg/μL, diluted in 2X lithium dodecyl sulfate (LDS, Thermo Fisher Scientific) buffer with reducers and heated at + 100 °C for 10 min. 15 μg of samples were loaded on a 12% Bis-TrisCriterion^™^ gel (Bio-Rad) and migrated in MOPS buffer for 1 h at 165 V on ice. After protein transfer on nitrocellulose sheets, migration and quality of the transfer were checked with a ponceau S staining. The membrane was saturated for 1 h at room temperature, and was then incubated with the AT100 (pT212-pS214, Life technologies MN1060), 2H9 (pS422, 4BioDx 4BDX-1501), tau-Nter (12–21, LB lab-made) or tau-Cter (clone 9F6, LB lab-made) antibodies overnight at + 4 °C. A peroxidase coupled secondary anti-rabbit or anti-mouse antibody was then applied for 45 min at room temperature. Immunoblotting was revealed by ECL. GAPDH (Sigma 9545) was used as a loading control. For Iba1 and GFAP evaluations, extracted samples were denatured at + 90 °C for 5 min in a buffer containing 1X LDS (NuPAGE^®^ LDS sample buffer, Invitrogen) and DTT 1X (NuPAGE^®^ sample reducing agent, Invitrogen). 10 µg of denatured protein were loaded per well. Samples and molecular weight marker (Bio-Rad Precision Plus Protein™ Dual Color standards) were loaded on 4–20% Criterion^™^ TGX^™^ gels (Bio-Rad) and migration was performed in a 1X tris–glycine buffer (Bio-Rad) at 120 V for 1 h. Proteins were then transferred to a nitrocellulose membrane using the Trans-Blot^®^ Turbo^™^ (Biorad) system. Migration and quality of the transfer were checked with a ponceau S staining. The membrane was then blocked with a TBS/0.1%Tween, 5% milk solution for 1 h at room temperature, and incubated with the primary antibody Iba1 (Wako 1919741, 1/2000), GFAP (Dako Z0334, 1/5000) or actin (Sigma A2066, 1/5000) diluted in saturation buffer overnight at + 4 °C. After washing in TBS/0.1%Tween solution, the membrane was incubated with the appropriate secondary HRP-conjugate antibody diluted to 1/5000 in TBS/0.1%Tween for 1 h at room temperature. The chemiluminescent signal was revealed using the Clarity western ECL (Bio-Rad) kit and the Chemidoc^™^ MP (Bio-Rad) imaging system. Protein band intensities were quantified on the ImageJ software and normalized by the actin expression level. For Aβ protein quantification, all assay-specific material (pre-coated microtiter plate, buffers, antibodies, standard solutions) was provided in the V-PLEX kit Aβ Peptide Panel 1 (6E10) (MSD^®^). Human brain homogenates were diluted to 1/5 (Ctrl samples) or 1/10 (AD samples) in the dilution buffer. As described in the manufacturer's protocol, the microtiter plate was blocked for 1 h at room temperature with the appropriate buffer. After washing, 25 µl of detection antibody and 25 µl of diluted sample or standard were added in duplicate to the wells and incubated under continuous agitation for 2 h at room temperature. Wells were washed and 150 µl of reading buffer was added. Plate reading was performed with the MSD Sector Imager 2400 (model 1200) multiplex assay system. Aβ_1–38_, Aβ_1–40_ and Aβ_1–42_ quantifications were performed with the Discovery Workbench 4.0 MSD^®^ software. Tau protein quantifications (total tau and phosphot-tau181) were performed according to the manufacturer’s protocol. Briefly, brain homogenates were diluted to 1/100 and 1/200 in the provided dilution buffer. 50 µl of standards or samples, as well as 50 µl of detection antibody solution were added to wells and incubated for 14 h at + 4 °C. After washing, 100 µl of 1X anti-rabbit IgG HRP solution was added for a 30 min incubation period at room temperature. 100 µl of stabilized chromogen were then added to each well for 30 min at room temperature, in the dark. The reaction was stopped by adding 100 µl of Stop solution and the plate was read at 450 nm within the hour. Data were analyzed with GraphPad Prism 7 using the 4PL method. All samples were tested in duplicates.

### Brain extracts preparation

Parietal cortex samples from each patient were individually homogenized at 10% weight/volume (w/v) in a sterile 1X Dulbecco’s phosphate buffer solution in CK14 soft tissue homogenizing tubes at 5000 rpm for 20 s (Precellys^®^, Bertin technologies). They were then sonicated on ice for 5 s at 40% amplitude and centrifuged at 3000 g for 5 min at + 4 °C. The resulting supernatant was aliquoted in sterile polypropylene tubes and stored at − 80 °C until use. Ten percent individual brain extracts were thawed on ice and combined together according to three groups: (i) Control (n = 2), (ii) AD patients with a disease duration of 5 to 8 years (n = 4, AD1), or (iii) AD patients with a disease duration of 6 months to 3 years (n = 4, AD2). Aβ levels, total tau and phospho-tau181 as well as Iba1 and GFAP protein levels were assessed by biochemistry in each combined brain extract.

### Transgenic mice

Mouse experiments used the APP_swe_/PS1_dE9_ mouse model of amyloidosis (C57Bl6 background) [8]. Aβ plaques can be detected as early as 4 months of age in these mice and increase in number and total area with age [8]. This model expresses endogenous murine tau protein isoforms and is not transgenic for any human tau. At the time of the inoculation of AD or Ctrl brain extracts, at 2 months of age, these mice did not have Aβ plaques. Animals were studied for four or eight months after intracerebral inoculation of the brain extracts (at 4 and 8 months post-inoculation (mpi) respectively, *n*_*Ctrl*_ = 11 and 15, *n*_*AD1*_ = 14 and 15, *n*_*AD2*_ = 12 and 20). Wild-type littermates injected with the Ctrl brain sample were used as controls for the behavioral tests (at 4 and 8 mpi respectively, *n*_*WT*_ = 6 and 12). A cohort of 21 animals was studied by immunohistochemistry one month post-inoculation (*n*_*Ctrl*_ = 6, *n*_*AD1*_ = 7, *n*_*AD2*_ = 8). As the Aβ plaques and tau loads were similar between AD1 and AD2 inoculated animals at a given time point (see results), measures from AD1 and AD2 animals were pooled within a single group that we called AD_be_-inoculated and brain extracts are referred to as AD_be_. All APP_swe_/PS1_dE9_ mice were born and bred in our center (Commissariat à l’Energie Atomique, centre de Fontenay-aux-Roses; European Institutions Agreement #B92-032-02). All animals were randomly assigned to the experimental groups using a simple procedure: They were identified using increasing numbers based on their birthdate. Animals with increasing numbers were alternatively assigned to the Ctrl (animal 1, 4, 7…), AD1 (animal 2, 5, 8…) and AD2 groups (animal 3, 6, 9…). Males were exclusively used in this study in order to optimize group homogeneity (Aβ plaque load is known to vary between males and females). Mice were injected during different inoculation sessions and each group was randomly inoculated at each session to avoid an "order of treatment" confounding effect. All experimental procedures were conducted in accordance with the European Community Council Directive 2010/63/UE and approved by local ethics committees (CEtEA-CEA DSV IdF N°44, France) and the French Ministry of Education and Research (A17_083 authorization), and in compliance with the 3R guidelines. Animal care was supervised by a dedicated in-house veterinarian and animal technicians. Human endpoints concerning untreatable continuous suffering signs and prostrations were taken into account and not reached during the study. Animals were housed under standard environmental conditions (12-h light–dark cycle, temperature: 22 ± 1 °C and humidity: 50%) with ad libitum access to food and water. The design and reporting of animal experiments were based on the ARRIVE reporting guidelines [7]. Sample size was based on previous experiments for Aβ induction in APP_swe_/PS1_dE9_ mice after inoculation of human brain (n = 8 at 4 mpi estimated with significance level of 5%, a power of 80%, and a two-sided test) [9] and increased to take into account uncertainties for new markers (tau lesion load, memory and synaptic changes). No animals were excluded from the study. SL was aware of initial group allocation, but further analyses (memory evaluations and post-mortem studies) were performed blinded.

### Stereotaxic surgery

Ten percent Ctrl, AD1 and AD2 brain extracts were thawed on ice. Extracts were then pooled together according to their group and the three resulting combined samples (Ctrl_be_, AD1_be_, AD2_be_) were sonicated (70% amplitude, 10 s on/off; Branson SFX 150 cell disruptor sonicator, 3.17 mm microtip probe Emerson, Bron) on ice in a sterile environment, extemporaneously before stereotaxic injection.

Two month-old APP_swe_/PS1_dE9_ and wild-type littermates were anesthetized by an intraperitoneal injection of ketamine (1 mg/10 g; Imalgène 1000, Merial) and xylazine (0.1 mg/10 g; 2% Rompun, Bayer Healthcare). Local anesthesia was also performed by a subcutaneous injection of lidocaine at the incision site (1 µl/g; 0.5% Xylovet, Ceva). Mice were placed in the stereotaxic frame (Phymep) and bilateral injections of brain samples were performed in the dorsal hippocampus (AP − 2 mm, DV − 2 mm, L ± 1 mm from bregma). Two µl/site of sample were administered using 34-gauge needles and Hamilton syringes, at a rate of 0.2 µl/min. After the injection, needles were kept in place for 5 more minutes before removal and the incision was sutured. The surgical area was cleaned before and after the procedure using povidone iodine (Vétédine, Vétoquinol). Respiration rate was monitored and body temperature was maintained at 37 ± 0.5 °C with a heating pad during the surgery. Anesthesia was reversed with a subcutaneous injection of atipamezole (0.25 mg/kg; Antisedan, Vetoquinol). Mice were placed in a ventilated heating box (25 °C) and monitored until full recovery from anesthesia. Postoperative anticipatory pain management consisted of paracetamol administration in drinking water (1.45 ml/20 ml of water; Doliprane, Sanofi) during 48 h.

### Behavioral evaluations

A novel object recognition task in a V-maze was used to investigate cognition at 4 mpi or 8 mpi on brain-extract inoculated APP_swe_/PS1_dE9_ mice. Wild-type littermates injected with the Ctrl_be_ were used as controls for the tests. Mice were handled for 2 min per day, for 5 days prior to any test to prevent stress effects during tasks. Prior to each test, mice were habituated to the experimental room for 30 min. The experimenter was blind to mouse groups. Performances were recorded using a tracking software (EthoVision XT14, Noldus).

The V-maze arena consisted of two 6 cm-wide, 33.5 cm-long and 15 cm-high black arms forming a V shape and exposed to 50 lx-lighting. The test was divided into three phases, each one separated by 24 h. At the beginning of each session, mice were placed at the center of the arena, i.e. at the intersection of the arms. During the habituation phase (day 1), mice were free to explore the empty arena for 9 min. The distance travelled was automatically recorded as an indicator of their exploratory activity. For the training phase (day 2), two identical objects (bicolor plastic balls) were placed at the end of each arm. Exploratory activity was evaluated as the time spent exploring the objects (manually recorded) and the distance travelled during the 9-min trial. On the test day (day 3), one familiar object (a bicolor plastic ball) was replaced by a novel one of a different shape and material (a transparent glass flask). Recognition was assessed using a discrimination index, calculated as follows:

$$Discrimination\,\, index= \frac{Time\,\, exploring\,\, the \,\,novel \,\,object-Time \,\,exploring\,\, the\,\, familiar \,\,object}{Total\,\, exploration\,\, time}$$

It reflects the time spent exploring each object, and therefore, the ability to discriminate a novel object from a familiar, previously explored one. A low discrimination index score reveals that mice spent less time exploring the new object, i.e. still had marked interest in the familiar object, and suggests that memory was impaired. Between each run, the V-maze was cleaned with 10% ethanol, effectively eliminating any scents from previous visites.

### Animal euthanasia and brain preparation for histology

Mice were sacrificed at 4 or 8 mpi, after the behavioral tests, with an intraperitoneal injection of a lethal dose of pentobarbital (100 mg/kg; Exagon, Axience). They were perfused intracardiacally with cold sterile 0.1 M PBS for 4 min, at a rate of 8 ml/min. The brain was extracted and post-fixed in 4% paraformaldehyde for 48 h at + 4 °C, transferred into a 15% sucrose solution for 24 h and in a 30% sucrose solution for 48 h at + 4 °C for cryoprotection. Serial coronal sections of 40 µm were performed with a microtome (SM2400, Leica Microsystem) and stored at − 20 °C in a storing solution (glycerol 30%, ethylene glycol 30%, distilled water 30%, phosphate buffer 10%). Free-floating sections were rinced in a 0.1 M PBS solution (10% Sigma-Aldrich^®^ phosphate buffer, 0.9% Sigma-Aldrich® NaCl, distilled water) before use. Washing and incubation steps were performed on a shaker at room temperature unless indicated otherwise.

### Immunohistochemistry for Aβ, tau, microglia and astroglia

Aβ deposits were evaluated using a 4G8 labelling. Tau was evaluated using labelling with AT8 directed against hyperphosphorylated tau and AT100 that binds to a conformational epitope including phosphorylated Thr212 and Ser214. Microglia were evaluated using Iba1 and CD68 antibodies. Astrocytes were stained with the GFAP antibody.

4G8 labelling was performed after pretreating brain sections with 70% formic acid (VWR®) for 20 min at room temperature. AT8 and AT100 labellings were performed after a pre-treatment with EDTA 1X citrate (Diagnostic BioSystems^®^) for 30 min at 95 °C. All tissues were then incubated in 30% hydrogen peroxide (Sigma-Aldrich^®^) diluted 1/100 for 20 min to inhibit endogenous peroxidases. Blocking of non-specific antigenic sites was achieved over 1 h using a 0.2% Triton X-100/0.1 M PBS (Sigma-Aldrich^®^) (PBST) solution containing 4.5% normal goat serum or 5% bovine serum albumin. Sections were then incubated at + 4 °C with the 4G8 (Biolegend 800706, 1/500), Iba1 (Wako 1919741, 1/1000), CD68 (Serotec—Biorad MCA 1957, 1/800) or GFAP (Dako Z0334, 1/10000) antibody diluted in a 3%NGS/PBST solution for 48 h, or with the AT8 (Thermo MN1020B, 1/500) or AT100 (Thermo MN1060, 1/500) antibody diluted in a 3%NGS/PBST solution for 96 h. After rinsing, an incubation with the appropriate biotinylated secondary antibody diluted to 1/1000 in PBST was performed for 1 h at room temperature, followed by a 1 h incubation at room temperature with a 1:250 dilution of an avidin–biotin complex solution (ABC Vectastain kit, Vector Laboratories^®^). Revelation was performed using the DAB Peroxidase Substrate Kit (DAB SK4100 kit, Vector Laboratories^®^). Sections were mounted on Superfrost Plus slides (Thermo-Scientific^®^). For the AT8 and AT100 labellings, a cresyl violet counterstain was performed. All sections were then dehydrated in successive baths of ethanol at 50°, 70°, 96° and 100° and in xylene. Slides were mounted with the Eukitt^®^ mounting medium (Chem-Lab^®^).

Stained sections were scanned using an Axio Scan.Z1 (Zeiss^®^—Z-stack images acquired at 20× (z-stacks with 16 planes, 1 µm steps with extended depth of focus)). Each slice was extracted individually in the .czi format using the Zen 2.0 (Zeiss^®^) software. Image processing and analysis were performed with the ImageJ software. Macros were developed for each staining in order to attain a reproducible semi-automated quantification. Images were imported with a 50% reduction in resolution (0.44 µm/pixel), converted to the RGB format and compressed in .tif format. For the 4G8, Iba1 and CD68 immunostainings, segmentation was performed through an automatic local thresholding using the Phansalkar method (radius = 15). Aβ load was evaluated after quantification of the 4G8-labelled particles between 8 and 2000 µm^2^, and normalization to the surface area of each ROI. Microglial status was evaluated as a percentage of Iba1- or CD68-positive surface area in each ROI. For the AT8 and AT100 stainings, the blue component of each image was extracted to remove the cresyl violet counter-staining from the analysis. AT8-positive or AT100-positive tau loads were assessed following an automatic local thresholding of the staining with the Phansalkar method followed by an evaluation of the percentage of AT8-positive or AT100-positive surface area in each ROI.

Tau lesions occur in the form of neuropil threads, neurofibrillary tangles (NFTs), and neuritic plaques i.e. tau aggregates within neurites surrounding Aβ deposits. In addition for the AT8 immunostaining, a quantification of neuritic plaques and NFTs was performed by manual counting. The AT8-positive area stained within neuritic plaques was evaluated by drawing circular regions of interest (with a constant area of 6 µm^2^), and by quantifying the percentage of tau-positive regions within each ROI, using the same thresholding method as previously described. A semi-quantitative analysis of neuropil threads was also performed by assigning a severity score based on the intensity and extent of AT8-positive staining in each ROI. All quantifications were performed on adjacent slices between − 0.34 and − 4.36 mm from bregma. Ten adjacent slices were analyzed for the 4G8 staining, and 5 for Iba1, CD68, AT8, and AT100 stainings. All ROIs were manually segmented using the Paxinos and Franklin neuro-anatomical atlas of mouse brain [20].

### Gallyas silver staining

Free-floating sections were mounted on Superfrost Plus (Thermo-Scientific^®^) slides and dried overnight prior to Gallyas staining. Section were permeabilized by successive incubations in toluene (2 × 5 min) followed by ethanol at 100°, 90° and 70° (2 min per solution). Slides were then incubated in a 0.25% potassium permanganate solution for 15 min, in 2% oxalic acid for 2 min then in a lanthanum nitrate solution (0.04 g/l lanthanum nitrate, 0.2 g/l sodium acetate, 10% H_2_O_2_ 30%) for 1 h to reduce non-specific background. Several rinses with distilled water were performed followed by an incubation in an alkaline silver iodide solution (3.5% AgNO_3_ 1%, 40 g/l NaOH, 100 g/l KI) for 2 min. The reaction was neutralized with 0.5% glacial acetic acid baths (3 × 1 min) and sections were incubated for 20 min in a developing solution (2 g/l NH_4_NO_3_, 2 g/l AgNO_3_, 10 g/l tungstosilicilic acid, 0.76% formaldehyde 37%, 50 g/l anhydrous Na_2_CO_3_). Several rinses with 0.5% acetic acid (3 × 1 min) followed by an incubation in 1% gold chloride solution for 5 min were then carried out. Sections were rinsed with distilled water and the staining was fixed with a 1% sodium thiosulfate solution. All sections were then rinsed with distilled water and dehydrated for 1 to 5 min in successive baths of ethanol at 50°, 70°, 96° and 100° and in xylene. Slides were mounted with the Eukitt^®^ mounting medium (Chem-Lab®). All steps were performed at room temperature.

### Co-stainings of microglia and Aβ plaques

In order to evaluate microglial load surrounding Aβ plaques, the co-staining of microglia and Aβ plaques was performed. Free-floating sections were permeabilized in a 0.2% Triton X-100/0.1 M PBS (Sigma-Aldrich^®^) solution for 3 × 10 min. Slices were stained by MXO4 dye (Tocris #4920, 1/300) for 30 min at room temperature, and then washed in a 0.1 M PBS solution. Sections were blocked in a 4.5%NGS/PBST solution for 1 h at room temperature before being incubated with the Iba1 antibody (Wako 1919741, 1/1000). 24 h later, sections were rinsed in 0.1 M PBS and incubated for 1 h at room temperature with the appropriate secondary antibody diluted to 1/1000 in PBST (anti-rabbit AlexaFluor 633). Sections were rinsed and mounted on Superfrost Plus (Thermo-Scientific^®^) slides with the Vectashield^®^ mounting medium with a refractive index of 1.45. Images of stained sections were acquired using a Leica DMI6000 confocal optical microscope (TCS SPE) with a 40 × oil-immersion objective (refractive index 1.518) and the Leica Las X software. A confocal zoom of 3 and a pinhole aperture fixed at 1 Airy were applied. Acquisition was performed in sequential mode with a sampling rate of 1024 × 1024 and a scanning speed of 700 Hz. Image resolution was 60 nm/pixel and the optical section was 0.896 µm. Twelve separate planes with a 0.1 µm step were acquired. The excitation wavelengths were 633 nm (for Iba1) or 350 nm (for Aβ). Image acquisition was performed on 2 slices located between − 3.28 and − 4.24 mm from the bregma, with 3 images per slice for the CA1 region and for the perirhinal/entorhinal cortex. 3D deconvolution of the images was performed using the AutoQuant X3 software. The deconvoluted 8-bit images were analyzed using the ImageJ software. Quantification of microglial load around plaques was based on a thresholding procedure applied across all images to segment microglial cells. MXO4-positive surfaces were dilated as circular regions of interest (with a diameter of 40 µm) drawn around the Aβ plaque to define a dilated plaque area. Microglial staining within the dilated surface, e.g. within the plaque area, was included in the analysis.

### Evaluation of synaptic density

Synaptic density was evaluated in the hippocampus (CA1) and the perirhinal/entorhinal cortex of all inoculated mice using a double immunolabelling of presynaptic (Bassoon) and postsynaptic (Homer1) markers. Free-floating sections were permeabilized in a 0.5% Triton X-100/0.1 M PBS (Sigma-Aldrich^®^) solution for 15 min. Slices were incubated with Bassoon (Abcam Ab82958, 1/200) and Homer1 (Synaptic systems 160003, 1/400) antibodies diluted in 3% BSA/PBST solution for 24 h at + 4 °C. Incubation with secondary antibodies coupled to a fluorochrome (Alexa Fluor) diluted in a 3% BSA/0.1 M PBS solution was then performed for 1 h at room temperature. Sections were rinsed and mounted on Superfrost Plus (Thermo-Scientific^®^) slides with the Vectashield^®^ mounting medium with a refractive index of 1.45. Images of stained sections were acquired using a Leica DMI6000 confocal optical microscope (TCS SPE) with a 63 × oil-immersion objective (refractive index 1.518) and the Leica Las X software. A confocal zoom of 3 and a pinhole aperture fixed at 1 Airy were applied. Acquisition was performed in sequential mode with a sampling rate of 1024 × 1024 and a scanning speed of 700 Hz. Image resolution was 60 nm/pixel and the optical section was 0.896 µm. 26 separate planes with a 0.2 µm step were acquired. The excitation wavelengths were 594 nm or 633 nm. Image acquisition in the CA1 region was performed on 4 adjacent slices located between − 1.82 and − 3.28 mm from the bregma, with 2 images per slice. For the perirhinal/entorhinal cortex, 3 adjacent slices located between − 3.28 and − 4.24 mm from the bregma were analyzed, with 2 images acquired per slice. 3D deconvolution of the images was performed using the AutoQuant X3 software. The deconvoluted 8-bit images were analyzed using the ImageJ software, as described in Gilles et al. [10]. Briefly, automated 3D segmentation of the presynaptic (Bassoon) and postsynaptic (Homer1) stained deconvoluted images was performed using "3D spots segmentation" from ImageJ (with "gaussian fit", "block" and "no watershed" options; https://imagej.net/plugins/3d-segmentation). Co-localization of overlapping objects was evaluated using "3D MultiColoc" from imageJ (https://imagejdocu.list.lu/plugin/analysis/details_about_multi-colocalisation_analysis/start). The number of colocalized objects was quantified as an index of synaptic density.

### Statistical analysis

Statistical analysis was performed using the GraphPad Prism software 8. For the behavioral tasks analysis, Kruskal–Wallis tests with Dunn’s multiple comparisons were perfomed except when repeated measures were acquired, in which case, a two-way repeated measures ANOVA with the Geisser-Greenhouse correction and Dunnett’s multiple comparisons was carried out. For the post-mortem analysis, Mann–Whitney tests were performed in order to compare differences between AD_be_- and Ctrl_be_-inoculated mice. For correlation studies, Spearman correlation test was performed. The significance level was set at *p* < 0.05. Data are shown on scattered dot plots with mean ± standard error of the mean (s.e.m).

## Results

### Characterization and inoculation of human brain homogenates

We prepared two brain homogenates from sporadic AD patients, with each homogenate consisting of a combination of four brain extracts from patients with a classical evolving form of the pathology (AD1) or a more rapidly evolving form (AD2). A third homogenate, considered as a control, was prepared from the brains of two non-demented individuals (Ctrl). The characteristics of the selected subjects are presented in Additional file 1: Table S1 and Additional file 1: Fig. S1. The amount of Aβ, tau and neuroinflammatory proteins slightly differed between the brain homogenates, as the AD2_be_ displayed more total tau and phospho-tau181, but less Aβ38 and Aβ40 than the AD1_be_ (Additional file 1: Fig. S1g–l). Iba1 and GFAP levels were similar in the two AD homogenates (Additional file 1: Fig. S1m–o).

### AD_be_-inoculated Aβ transgenic mice develop memory alteration

**Fig. 1:**
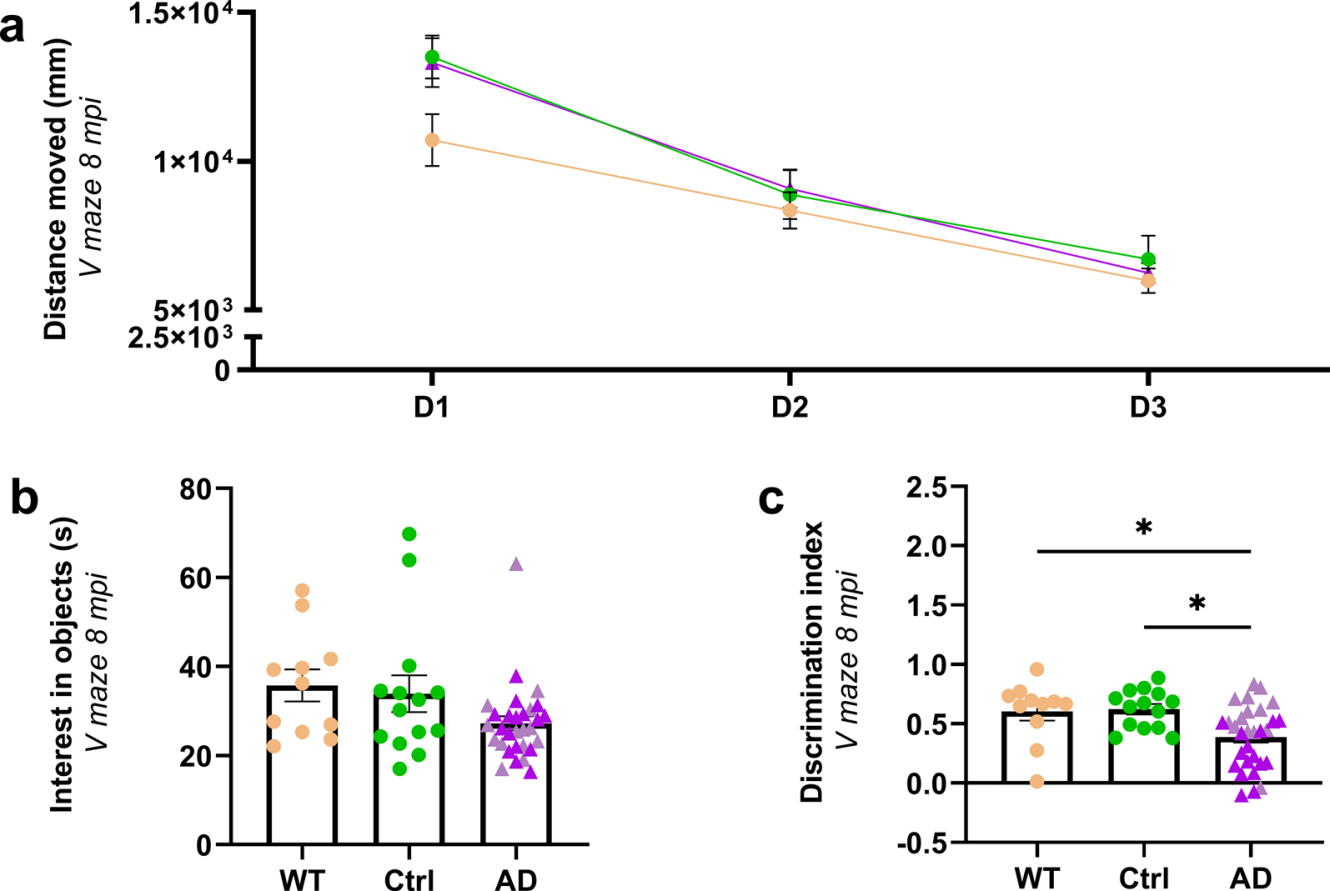
Object recognition deficits in the AD_be_-inoculated mice. Object recognition performances were evaluated at 8 mpi in a V-maze. WT mice and APP_swe_/PS1_dE9_ mice inoculated with Ctrl_be_ or AD_be_ had comparable exploratory activity, as suggested by the distance moved throughout the 3-day test (**a**, for the days: *F*_*(1.7, 90.5)*_ = 72.4, *p* < 0.0001; for the groups: *F*_*(2, 52)*_ = 1.2, *p* = 0.3; two-way repeated measures ANOVA with the Geisser-Greenhouse correction and Dunnett’s multiple comparisons) and the time spent on exploring the objects (**b**, *p* > 0.05; Kruskal–Wallis with Dunn’s multiple comparisons). During the novel object recognition evaluation, AD_be_-inoculated mice spent less time exploring the novel object, as suggested by a lower discrimination index, compared to WT and APP_swe_/PS1_dE9_ Ctrl_be_-inoculated mice (**c**, respectively, *p* = 0.027, and 0.016; Kruskal–Wallis with Dunn’s multiple comparisons). *n*_*Ctrl*_ = 15, *n*_*AD1*_ = 15 (light pink in **b**, **c**), *n*_*AD2*_ = 20 (dark pink in **b**, **c**), *n*_*WT*_ = 12 mice. *p < 0.05; Data are shown as mean ± s.e.m

AD_be_ and Ctrl_be_ were inoculated bilaterally in the dorsal hippocampus (CA1) of 2-month-old APP_swe_/PS1_dE9_ mice. An additional group of Ctrl_be_-inoculated wild-type littermates was used as an Aβ plaque-free control group. The mice were evaluated in an object-recognition task (V-maze test) at 8 mpi. Animals from each group showed similar exploratory activity [similar distance travelled throughout the three days of test (Fig. 1a) and comparable interest in the two identical objects during the training phase of the task (Fig. 1b)]. During the memory task, i.e. the novel object recognition evaluation, Ctrl_be_-inoculated Aβ plaque-free wild-type and Aβ plaque-bearing APP_swe_/PS1_dE9_ mice had similar performances (Fig. 1c). This suggests that Aβ plaques did not modulate memory in this task. AD_be_-inoculated APP_swe_/PS1_dE9_ animals mice spent less time exploring the novel object than Ctrl_be_-inoculated mice. This suggests that AD_be_ infusion induces memory impairment (Fig. 1c). Another cohort of mice was also evaluated at 4 mpi. No difference in memory performance was observed at this stage (Additional file 1: Fig. S2a–c).

### AD_be_-infusion increases Aβ plaque deposition and tau pathologies in proximity to the infusion site

**Fig. 2:**
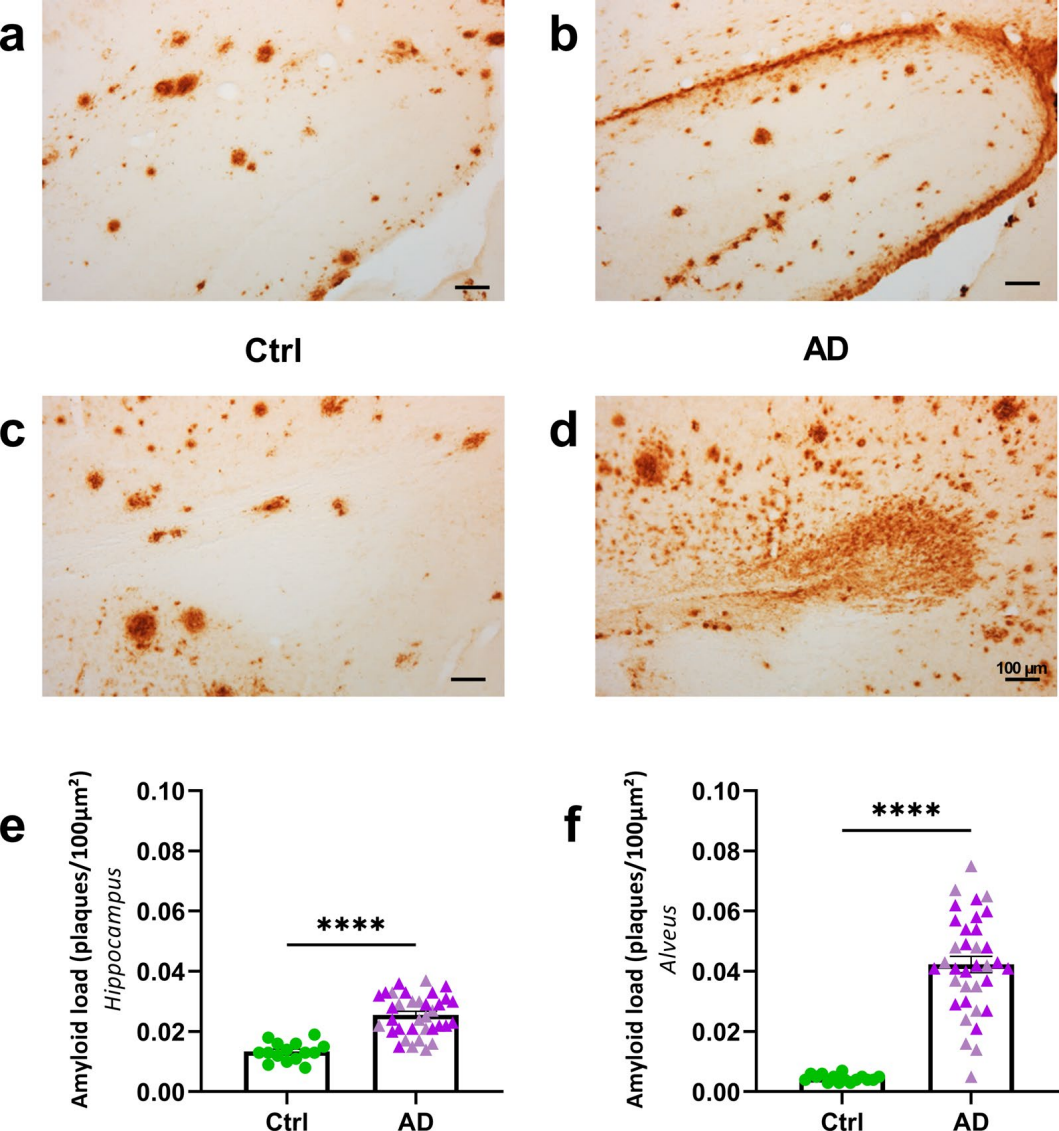
AD_be_ inoculation accelerates amyloidosis in APP_swe_/PS1_dE9_ mice in proximity to the injection site. **a**–**d** Representative images of 4G8 immunolabelling showing Aβ pathology in the hippocampus (**a**, **b**) and alveus (**c**, **d**) of APP_swe_/PS1_dE9_ mice eight months after human brain inoculation. **e**, **f** Quantification of Aβ load (4G8-positive Aβ plaques per 100 µm^2^) revealed that AD_be_ inoculation accelerates Aβ deposition in the hippocampus (**e**, Mann Whitney's test, U = 21.5, *p* < 0.0001) and alveus (**f**, Mann Whitney's test, U = 5, *p* < 0.0001). ****p < 0.0001. *n*_*Ctrl*_ = 15, *n*_*AD1*_ = 15 (light pink in **e**, **f**), *n*_*AD2*_ = 20 mice (dark pink in **e**, **f**). Data are shown as mean ± s.e.m. Scale bars = 100 µm

At 8 mpi, infusion of AD_be_ led to an increased Aβ plaque deposition in the hippocampus and in the region surrounding the alveus compared to Ctrl_be_ (Fig. 2). This increase in Aβ pathology already started at 4 mpi (Additional file 1: Fig. S2d, e).

**Fig. 3:**
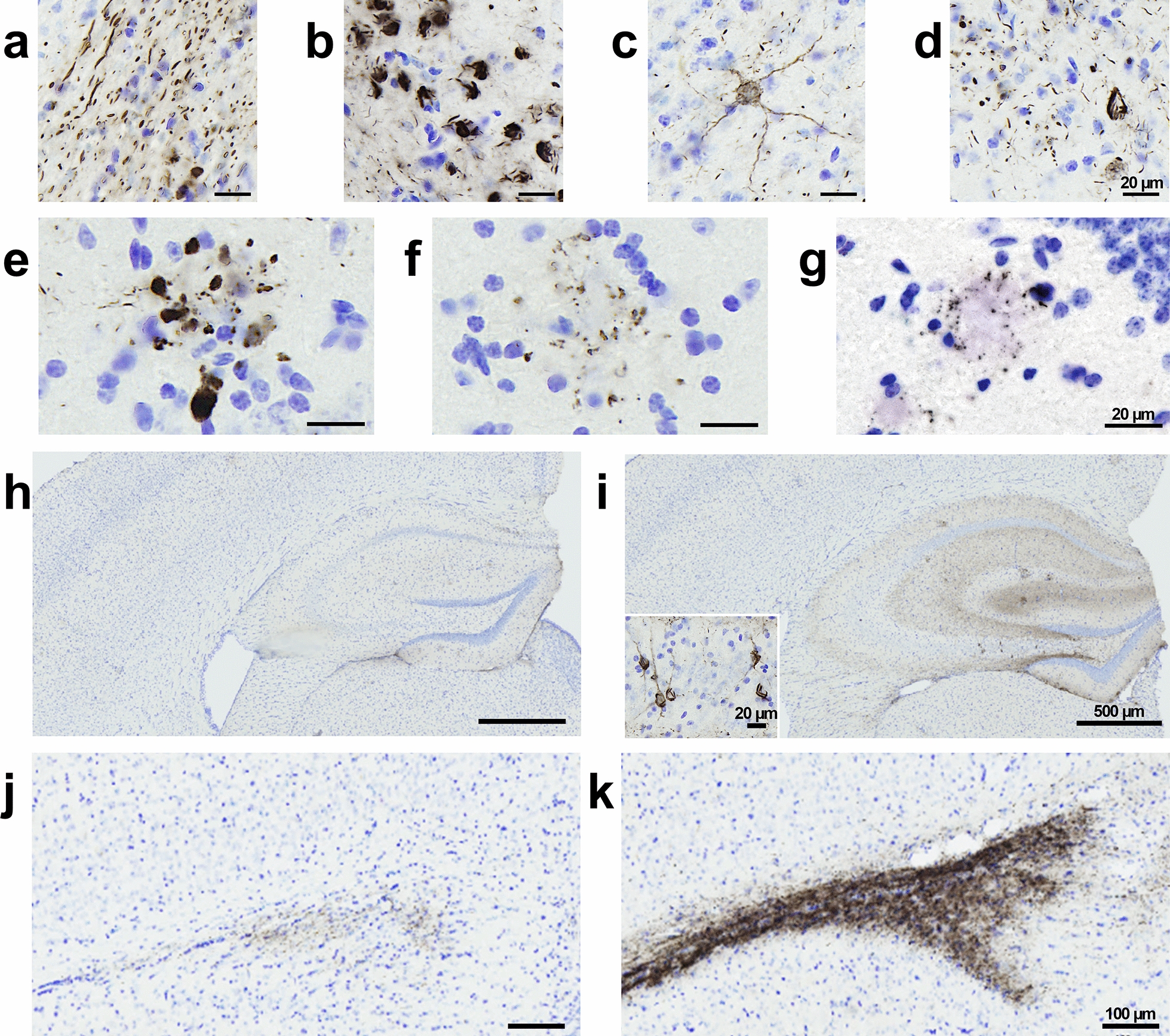
AD_be_ inoculation increases tau pathology in APP_swe_/PS1_dE9_ mice in proximity to to the injection site. AT8 immunolabelling revealed tau lesions in the forms of neuropil threads (**a**), NFTs (**b**–**d**), neuritic plaques (**e**) around the injection site of AD_be_-inoculated APP_swe_/PS1_dE9_ mice at 8 mpi. Neuritic plaques were also detected in Ctrl_be_-inoculated mice at 8 mpi (**f**). AT8 immunolabelling of non-inoculated aged APP_swe/_PS1_dE9_ mice (24 months old) also revealed neuritic plaques (**g**). **h**, **i** Representative images of AT8 immunolabelling showing tau pathology induction in the dorsal hippocampus of an AD_be_-inoculated APP_swe_/PS1_dE9_ mouse at 8 mpi (**i**) compared to a Ctrl_be_-inoculated littermate (**h**). **j**, **k** Representative images of AT8 immunolabelling showing tau pathology induction in the alveus of an AD_be_-inoculated APP_swe_/PS1_dE9_ mouse at 8 mpi (**k**) compared to a Ctrl_be_-inoculated littermate (**j**). Scale bars = 20 µm (**a–g**), 500 µm (**h**, **i**) and 100 µm (**j**, **k**) and 20 µm (**i** inset)

In humans, tau lesions occur in the form of neuropil threads, NFTs and neuritic plaques i.e. tau aggregates within neurites surrounding Aβ deposits. These three lesion types were detected in the hippocampus of AD_be_-inoculated APP_swe_/PS1_dE9_ mice at 4 and 8 mpi [Fig. 3a: neuropil threads (NTs); Fig. 3b–d: NFTs; Fig. 3e: AT8-positive neuritic plaques (NPs)]. At 1 mpi, tau lesions were not detected in any animal (Additional file 1: Fig. S3).

**Fig. 4:**
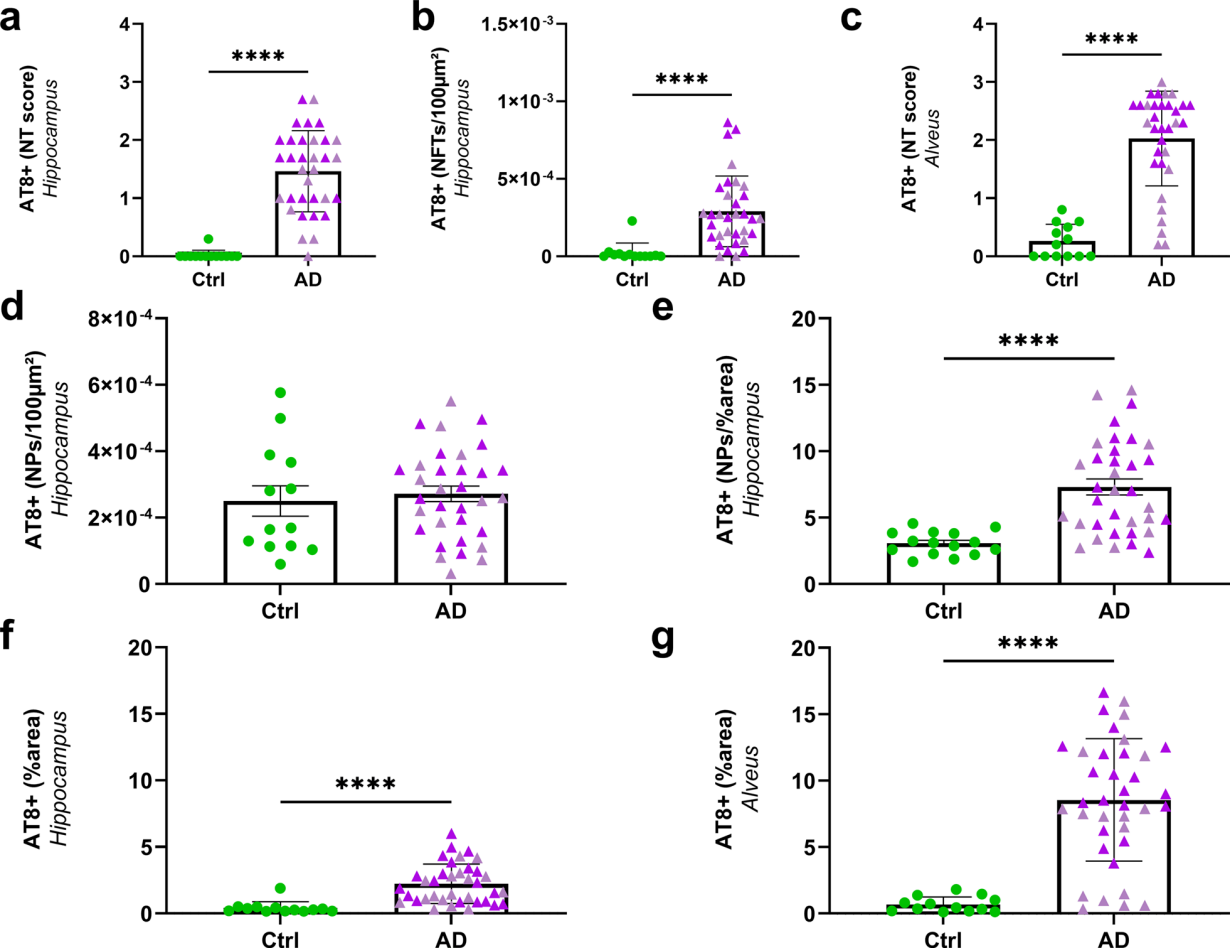
AD_be_ inoculation increases tau pathology in APP_swe_/PS1_dE9_ mice close to the injection site (8 mpi). Increased AT8-positive neuropil threads (**a**, *p* < 0.0001) and NFTs (**b**, AT8-positive NFTs per 100 µm^2^ of area, *p* < 0.0001) in the hippocampus as well as neuropil threads in the alveus (**c**, *p* < 0.0001) of AD_be_-inoculated animals compared to Ctrl_be_-animals. The number of AT8-positive neuritic plaques was similar in all groups (NPs per 100 µm^2^ of area, **d**), but the AT8-positive area within neuritic plaques was higher in AD_be_- compared to Ctrl_be_-inoculated animals (**e**, *p* < 0.0001). Increased overall AT8-labelled phospho-tau in the hippocampus (**f**) and alveus (**g**) following AD_be_ inoculation compared to Ctrl_be_ (percentage of AT8-positive area, *p* < 0.0001). ****p < 0.0001. *n*_*Ctrl*_ = 15, *n*_*AD1*_ = 15 (light pink), *n*_*AD2*_ = 20 mice (dark pink). Mann Whitney's tests. Data are shown as mean ± s.e.m. NT = neuropil threads; NFT = neurofibrillary tangles; NP = neuritic plaques

These three tau lesions did not occur similarly in AD_be_- and Ctrl_be_-inoculated APP_swe_/PS1_dE9_ mice. AT8-positive neuropil threads or NFTs were detected in the hippocampus of AD_be_-inoculated mice but not of Ctrl_be_-inoculated animals (Fig. 4a, b). Neuropil threads were also detected in the alveus of AD_be_- but not of Ctrl_be_-inoculated animals (Fig. 4c).

Unlike neuropil threads or NFTs, neuritic plaques were detected in both AD_be_- (Fig. 3e) and Ctrl_be_-inoculated (Fig. 3f) APP_swe_/PS1_dE9_ mice at 8 mpi and the number of neuritic plaques was similar in their hippocampus (Fig. 4d). The AT8-positive area stained within neuritic plaques was however larger in the AD_be_-inoculated groups compared to Ctrl_be_ animals (Fig. 4e). To investigate whether human brain extract inoculation was necessary to induce neuritic plaques, we performed an AT8 immunostaining on 24-month-old non-inoculated APP_swe_/PS1_dE9_ mice and observed neuritic plaques (Fig. 3g). Thus AT8-positive neuritic plaques occur spontaneously in APP_swe_/PS1_dE9_ mice.

Further quantitative analysis at 8 mpi showed that, compared to Ctrl_be_-inoculated mice, AD_be_-inoculated animals displayed an increase in AT8-positive tau lesions in the hippocampus (Figs. 3h, i, 4f) and in the alveus (Figs. 3j, k, 4g). This increase was already reported at 4 mpi, although to a lesser extent (Additional file 1: Fig. S2g, h).

AT100 detects late stage phospho-epitope found mostly in intracellular NFTs in humans and is used as a marker of aggregation [5]. Within the hippocampus, it labelled neuropil threads and NFTs in the alveus part of AD_be_-inoculated mice (Additional file 1: Fig. S4a, b). AT100 staining was increased in the alveus of AD_be_-inoculated mice but not in the other parts of the hippocampus (Additional file 1: Fig. S4c, d).

Gallyas silver staining is used for the detection of aggregated tau pathology and paired helical filament-specific argentophilic tau lesions within neurofibrillary tangles. It revealed neuropil threads (Additional file 1: Fig. S4e), as well as Aβ plaques (Additional file 1: Fig. S4f) in AD_be_-inoculated mice.

### Spreading of tau pathology

**Fig. 5:**
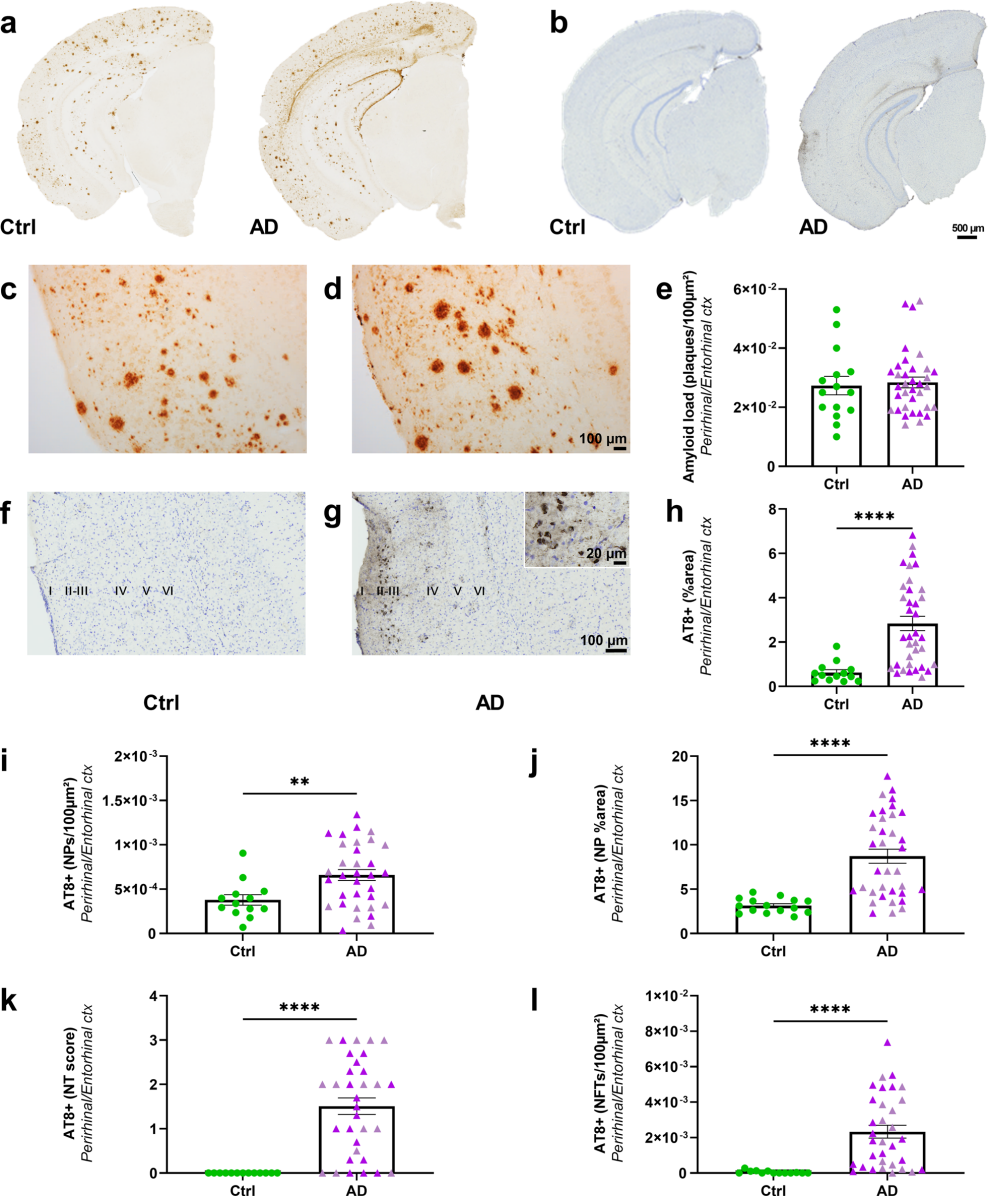
Aβ and tau pathologies in the perirhinal/entorhinal cortex following AD_be_ inoculation in APP_swe_/PS1_dE9_ mice (8 mpi). Representative images of the 4G8 immunolabelling showing Aβ pathology (**a**) and the AT8 immunolabelling showing tau pathology (**b**) in Ctrl_be_- and AD_be_-inoculated APP_swe_/PS1_dE9_ mice. Magnified views of Aβ (**c**, **d**) and tau (**f**, **g**) lesions in the perirhinal/entorhinal cortex. Quantification of Aβ load (**e**, *p* > 0.05), overall AT8-positive tau lesions (**h**, *p* < 0.0001), neuritic plaque (NPs) count (**i**, *p* = 0.0098), AT8-positive area stained within neuritic plaques (**j**, *p* < 0.0001), neuropil threads (NTs—**k**, *p* < 0.0001), and NFTs (**l**, *p* < 0.0001) in the perirhinal/entorhinal cortex. Mann Whitney's tests. **p < 0.01; ****p < 0.0001. *n*_*Ctrl*_ = 15, *n*_*AD1*_ = 15 (light pink), *n*_*AD2*_ = 20 mice (dark pink). Data are shown as mean ± s.e.m. Scale bars = 500 µm (**a**, **b**), 100 µm (**c**, **d, f**, **g**) and 20 µm in inset

The perirhinal/entorhinal cortex is connected to the hippocampus. In this region, Aβ load was similar in AD_be_- and Ctrl_be_-inoculated APP_swe_/PS1_dE9_ mice at 8 mpi (Fig. 5a, c–e) and at 4 mpi (Additional file 1: Fig. S2f). Aβ detected in this region may thus only reflect the endogenous expression of the peptide in the APP_swe_/PS1_dE9_ model.

Tau pathology was detected in the perirhinal/entorhinal cortex, mainly in the external layers II and III of the cortex (Fig. 5b, f, g) that project to the dentate gyrus via the perforant pathway and to the CA1 region via the temporo-ammonic pathway, respectively. Internal layers (e.g. layers V–VI) that receive projections from the CA1 region were not labelled. At 8 mpi, areas of AT8-positive tau lesions were increased in the perirhinal/entorhinal cortex of AD_be_-inoculated mice compared to Ctrl_be_-inoculated ones (Fig. 5h). As for the hippocampus, the three categories of tau lesions did not occur similarly in AD_be_- and Ctrl_be_-inoculated APP_swe_/PS1_dE9_ mice. Indeed, neuritic plaques were detected in both AD_be_- and Ctrl_be_-inoculated mice. However, the number of neuritic plaques (Fig. 5i) and the AT8-positive area stained within these neuritic plaques (Fig. 5j) were increased in AD_be_-inoculated compared to Ctrl_be_-inoculated mice. On the contrary, neuropil threads and NFTs only occurred in AD_be_ animals (Fig. 5k, l).

Other cortical regions such as the visual cortex also displayed neuropil threads, NFTs and neuritic plaques in AD_be_-inoculated mice (Additional file 1: Fig. S5a, b).

At 4 mpi, AT8-positive tau lesion loads were not different in the perirhinal/entorhinal cortex of AD_be_- and Ctrl_be_-inoculated mice (Additional file 1: Fig. S2i). The increased presence of tau in this region at 8 mpi in AD_be_-inoculated mice, thus reflects a time-dependent tau pathology spreading from 4 to 8 mpi.

AT100-positive tau lesions (Additional file 1: Fig. S6a–c) and Gallyas-positive (Additional file 1: Fig. S6d, e) tau lesions were detected in the perirhinal/entorhinal cortex of AD_be_-inoculated mice. Gallyas staining also revealed Aβ plaques (Additional file 1: Fig. S6d, f) in this region.

### Synaptic density is correlated with neuritic tau pathology but not with Aβ plaque load

**Fig. 6:**
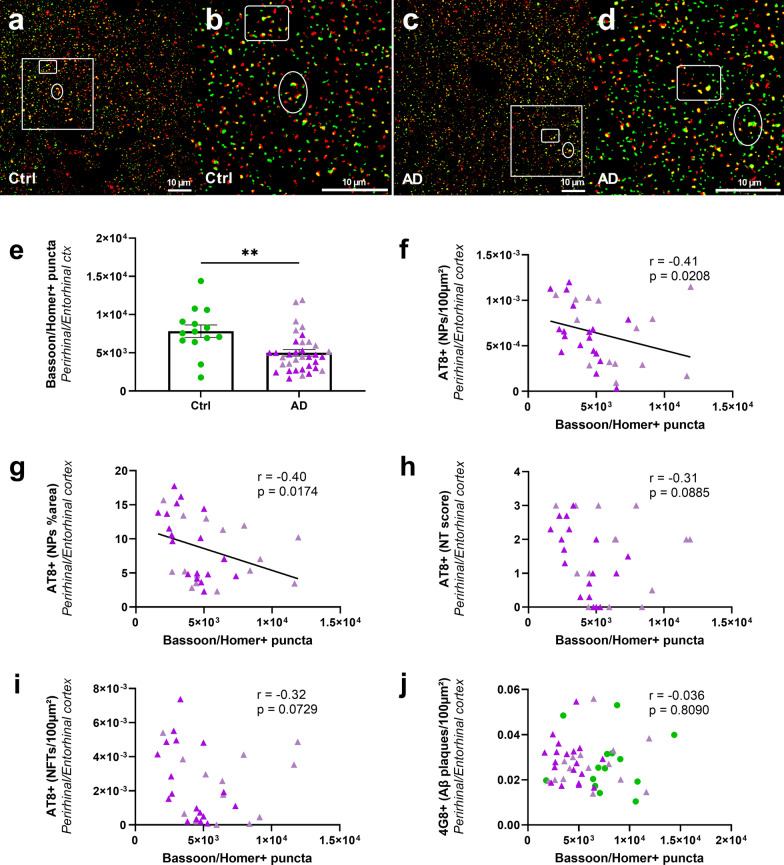
Synaptic density is decreased in AD_be_-inoculated APP_swe_/PS1_dE9_ mice and correlates with tau pathology in neuritic plaques (8 mpi). Representative image of Bassoon (red) and Homer1 (green) immunolabelling in Ctrl_be_- (**a**, **b**) and AD_be_-inoculated (**c**, **d**) animals. (non-segmented confocal optical microscopic images (**a**, **c**); segmented images of insets (**b**, **d**). Bassoon/Homer-positive puncta (yellow in **a**–**d**) were quantified as an index of synaptic density. The synaptic density decreased in perirhinal/entorhinal cortex of AD_be_ mice compared to Ctrl_be_-inoculated mice at 8 mpi (**e**, *p* = 0.002). Synaptic density was inversely correlated with tau pathology in neuritic plaques in the perirhinal/entorhinal cortex (**f**: neuritic plaque count; **g**: AT8-positive area within neuritic plaques), but not with neuropil threads (**h**), neurofibrillary tangles (**i**), or Aβ pathology (**j**). Spearman’s correlations with significance level at *p* < 0.05. *n*_*Ctrl*_ = 15, *n*_*AD1*_ = 15 (light pink), *n*_*AD2*_ = 20 mice (dark pink). **p < 0.01; Data are shown as mean ± s.e.m

Synaptic density was decreased by 36% in the perirhinal/entorhinal cortex (Fig. 6e) and 19% in the CA1 of the hippocampus (Additional file 1: Fig. S7) of AD_be_-inoculated mice compared to Ctrl_be_ mice at 8 mpi. It was not changed at 4 mpi (Additional file 1: Fig. S2j, k). We focused on the relationships between synaptic density and Aβ or tau lesions in the perirhinal/entorhinal cortex, i.e. at distance of the inoculation site at 8 mpi. The synaptic density was inversely correlated with neuritic plaque count (Fig. 6f) and the AT8-positive area stained within neuritic plaques (Fig. 6g). Other tau-positive lesions [neuropil threads (Fig. 6h) or NFTs (Fig. 6i)] were not correlated with synaptic density. Furthermore, synaptic density was not correlated with Aβ load (Fig. 6j).

### Synaptic impairments are correlated with reduced microglial activation

**Fig. 7:**
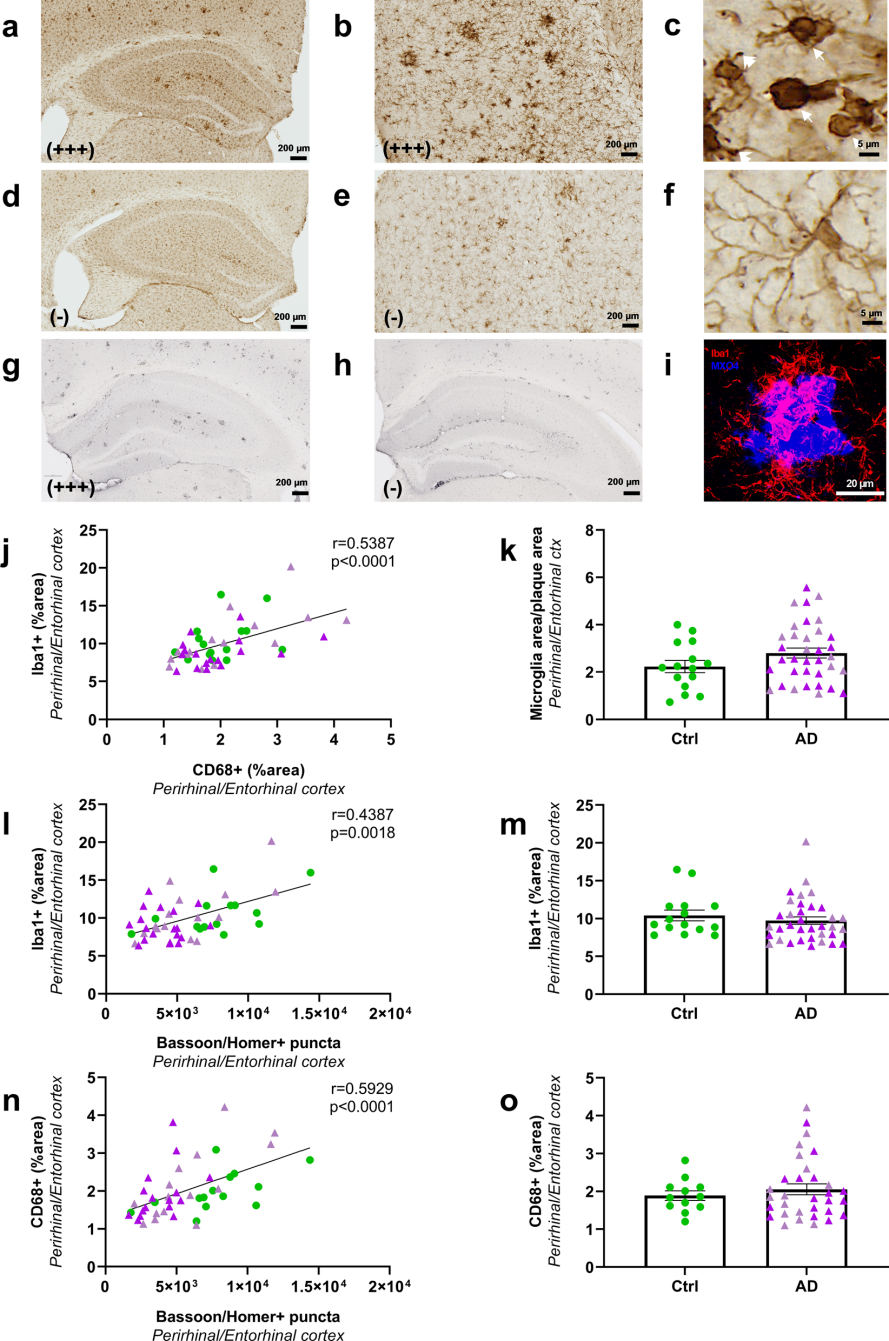
Microglial activation in AD_be_ and Ctrl_be_-inoculated APP_swe_/PS1_dE9_ mice (8 mpi). Within each group, animals with high and low Iba1 staining were identified. Representative images of one animal with higher Iba1 levels showing abundant staining in the hippocampus (**a**) and perirhinal/entorhinal cortex (**b**). Animals with higher Iba1 levels displayed activated microglia characterized by beading with spheroidal swellings of the processes and enlarged cell body with dystrophic ramifications (**c**, arrows). Animals with lower Iba1 levels (**d**, **e**) displayed more cells with a highly ramified profile (**f**) consistent with a non-activated phenotype. Representative images of one animal with higher (**g**) or lower (**h**) CD68 staining in the hippocampus. Iba1 staining level was correlated with CD68 labelling in the perirhinal/entorhinal cortex (**j**). Similar levels of Iba1 (**m**) and CD68 (**o**) stained areas in the perirhinal/entorhinal cortex of Ctrl_be_ and AD_be_ animals (Mann Whitney's tests). Confocal microscopy showed Iba1-positive microglia (red) surrounding Aβ deposition stained by MXO4 (blue) (**i**). The Iba1-positive microglial load surrounding the Aβ plaques was similar in the different groups (**k**, Mann Whitney's test). Interestingly, synaptic density was positively correlated with microglial Iba1 (**l**) or CD68 (**n**) labelling in the perirhinal/entorhinal cortex (Spearman’s correlations). *n*_*Ctrl*_ = 15, *n*_*AD1*_ = 15 (light pink), *n*_*AD2*_ = 20 mice (dark pink). Scale bar = 20 µm

Neuroinflammation was evaluated as a complementary endpoint. It was assessed by staining brain tissues using Iba1 antibody (Fig. 7a–f), a general marker for microglia as well as an anti-CD68 antibody (Fig. 7g, h) a marker of phagocytic microglia that stains a lysosomal protein expressed at high levels by activated microglia. Visual observation of the Iba1 stained sections suggested different levels of staining with some animals displaying high labelling (Fig. 7a, b) with abundant microglia with an activated phenotype characterized by beading with spheroidal swellings and enlarged cell body with dystrophic ramifications (Fig. 7c, arrows) [24]. Some other animals had lower staining (Fig. 7d, e) with highly ramified microglia (Fig. 7f). Interindividual difference was also detected for CD68 labelling, with some animals displaying high labelling (Fig. 7g) and some others lower staining (Fig. 7h). Iba1 staining was positively correlated with CD68 staining in the hippocampus (r = 0.39, *p* = 0.009; not shown) or the perirhinal/entorhinal cortex (Fig. 7j).

AD_be_ and Ctrl_be_-inoculated mice had similar levels of Iba1 and CD68 stained areas in the hippocampus (not shown) or perirhinal/entorhinal cortex (Fig. 7m, o). Microglia are known to surround Aβ plaques where their activation shields neurons from Aβ plaque toxicity [4]. As expected, using confocal microscopy, we could detect Iba1-stained microglia surrounding Aβ plaques (Fig. 7i). We could not detect any differences in Iba1 staining around plaques in the different groups (Fig. 7k). This suggests no difference in the shielding effect in our inoculated groups. As for tau and Aβ, at 8 mpi, we further evaluated relationships between synaptic density and microglia in the perirhinal/entorhinal cortex, i.e. away from the inoculation site. A positive correlation was reported between synaptic density and Iba1 (Fig. 7l) or CD68 stainings (Fig. 7n).

Astrocyte reactivity (GFAP staining) was evaluated and no difference was detected between the groups in the hippocampus or in the perirhinal/entorhinal cortex (Additional file 1: Fig. S8). Additionally, synaptic density was not correlated with astrocyte-associated GFAP staining (not shown).

## Discussion

We infused AD_be_ into the brain of APP_swe_/PS1_dE9_ Aβ plaque-bearing mice. This (i) increased Aβ pathology in the vicinity to the inoculation site, (ii) induced tau lesions that spread in the brain, and (iii) induced downstream events including memory impairment and reduction of synaptic density. The time course of the induced pathology is reported at 1, 4 and 8 mpi (Additional file 1: Fig. S9).

The local increase of Aβ pathology close to the inoculation site is consistent with several studies [13, 17]. Aβ plaque spreading has been described following the inoculation of Aβ-enriched brain extracts from mice [31]. We did not reproduce these latter results as, unlike tau lesions, Aβ did not spread to the perirhinal/entorhinal cortex. This spreading effect might be hidden in our APP_swe_/PS1_de9_ mice that spontaneously develop high Aβ plaque load over time.

Tau lesions were detected in AD_be_-inoculated animals. The lack of tau detection at one month post-inoculation and its progressive occurrence thereafter suggests that tau detected at 4 months and later does not reflect a direct deposit of human tau from AD_be_ and that it is created from mouse tau.

Neuritic plaques, but not the other tau-lesions (neuropil threads and NFTs), were detected in Ctrl_be_-inoculated animals as well as in aged non-inoculated APP_swe_/PS1_dE9_ mice. Thus Aβ aggregates, per se, are sufficient to induce tau lesions in surrounding neurites leading to neuritic plaques as reported in previous studies [16, 19, 22]. The number of neuritic plaques was not increased at the inoculation site in AD_be_-inoculated animals, while the density of tau lesions within neuritic plaques was increased. This suggests that AD_be_ inoculation worsened the already present neuritic pathology.

Unlike neuritic plaques, neuropil threads and NFTs were only induced in AD_be_-inoculated animals. Two sources of tau seeds were available in AD_be_-inoculated animals: Endogenous murine tau issued from neuritic plaques (detected in both Ctrl_be_- and AD_be_-inoculated animals), and exogenous tau seeds issued from human AD_be_. Due to the presence of neuropil threads and NFTs only in AD_be_-inoculated animals, we hypothesize that exogenous tau from AD_be_ are the main source of seeds for tau induction and spreading in neuropil threads and NFTs. Consistent with this hypothesis, in a previous study, we induced neuropil threads and NFTs in primates by infusing the AD_be_ used in the present study [14].

The hippocampus, i.e. the inoculated regions, is connected to the perirhinal/entorhinal cortex. In this latter region, both the number of neuritic plaques and the AT8-positive area stained within these neuritic plaques were increased in AD_be_-inoculated animals. This suggests that increased tau lesions from the hippocampus participated to increase neuritic plaque occurrence and severity.

Synaptic impairment has never been evaluated in models with Aβ and tau pathology induced by inoculation of human brain extracts. Tau pathology within neuritic plaques (plaque counts or AT8-positive area) in the perirhinal/entorhinal cortex, i.e. away from the inoculation site, was correlated with synaptic deficits while Aβ plaques or tau pathology within neuropil threads or NFTs were not correlated with synaptic changes. These results, in a model that is not based on tau overexpression, strongly supports the hypothesis that neuritic tau pathology contributes to synaptic impairment. Whilst our results suggest tau toxicity, they do not rule out a possible toxic role of Aβ on synaptic impairment. Indeed, AD_be_- and Ctrl_be_-inoculated mice from our study had similar Aβ plaque loads in the perirhinal/entorhinal cortex, thus the Aβ plaque load effect can not be determined in our experimental set-up.

Memory evaluations did not show any difference between Ctrl_be_-inoculated Aβ plaque-free wild-type and Aβ plaque-bearing APP_swe_/PS1_de9_ mice at 8 mpi, i.e. at an age when Aβ plaques are numerous. This suggests a limited effect of Aβ plaques on memory at 8 mpi. On the contrary, AD_be_-inoculated animals that displayed tau lesions had memory impairments, which suggests a major impact of tau on the occurrence of cognitive changes. As Aβ and tau have been proposed to interact to induce memory changes [23], it is also possible that increasing tau pathology in mice increased Aβ impact on cognition.

Activation of microglia was evaluated as a complementary measure. Although we did not detect differences of microglia load in AD_be_- and Ctrl_be_-inoculated animals, we decided to assess local relationships between microglia and synapses in the perirhinal/entorhinal cortex i.e. away from the inoculation site, in regions that did not receive the inoculates of brain extracts. Unexpectedly, we found a correlation between synaptic loss and a reduction of microgliosis (for both Iba1 and CD68 stainings). This is the first observation of a relationship between microglial activation and synapse density in a mouse model with Aβ and tau lesions without genetic or chemical manipulation of microglia. One possible interpretation is that the toxic process leading to synaptic loss also impaired microglia. This hypothesis is supported by previous studies showing that tau pathology can drive microglial degeneration [24, 28]. As we found a relationship between neuritic tau pathology and synaptic deficit, we propose the hypothesis that tau could be the toxic species. Other hypothesis can however, also be proposed. For example, as soluble forms of Aβ are implicated in synaptotoxicity [25–27] and as Aβ can modulate microglial activity, Aβ could also be the toxic species. These hypotheses need now to be formally tested in future studies.

## Conclusions

Intracerebral infusion of AD_be_- into Aβ plaque–bearing mice that do not overexpress tau induced memory and synaptic impairments, increased Aβ load at the inoculation site as well as tau lesions that spread into connected areas. Based on our results, we thus propose the following sequence of events concerning the pathological changes of AD _be_-inoculated mice. Aβ plaques can induce tau lesions within neuritic plaques. In the presence of exogenous tau seeds issued from human brain extracts, the tau pathology is amplified and leads to neuropil threads and NFTs. These events favor the cerebral spreading of tau pathology in the form of neuritic plaques, neuropil threads and NFTs. Amongst the varied tau lesions, the neuritic plaques in particular, induce a reaction that is associated with synaptic density reduction. Finally, our data suggest that microglia regulates or is regulated by events leading to synaptic reduction. Further experiments will elucidate this complex sequence of events.

## Supplementary Information

**Additional file 1.** The additional file contains Table S1 and Figures S1-S9.

## Data Availability

Data that support the findings of this study are available from the corresponding author, upon request.

## Abbreviations

Aβ: Amyloid-β
AD: Alzheimer's disease
AD_be_: AD brain extracts
Ctrl: Control
Ctrl_be_: Control brain extracts
mpi: Months post-inoculation
NFTs: Neurofibrillary tangles
NP: Neuritic plaques
NT: Neuropil threads
WT: Wild-type

## References

[1] Audouard E, Houben S, Masaracchia C, Yilmaz Z, Suain V, Authelet M, De Decker R, Buee L, Boom A, Leroy K, Ando K, Brion JP (2016). High-molecular-weight paired helical filaments from Alzheimer brain induces seeding of wild-type mouse tau into an argyrophilic 4R Tau pathology in vivo. Am J Pathol.

[2] Bennett DA, Schneider JA, Wilson RS, Bienias JL, Arnold SE (2004). Neurofibrillary tangles mediate the association of amyloid load with clinical Alzheimer disease and level of cognitive function. Arch Neurol.

[3] Braak H, Braak E (1991). Neuropathological stageing of Alzheimer related changes. Acta Neuropathol.

[4] Condello C, Yuan P, Schain A, Grutzendler J (2015). Microglia constitute a barrier that prevents neurotoxic protofibrillar Abeta42 hotspots around plaques. Nat Commun.

[5] d'Orange M, Auregan G, Cheramy D, Gaudin-Guerif M, Lieger S, Guillermier M, Stimmer L, Josephine C, Herard AS, Gaillard MC, Petit F, Kiessling MC, Schmitz C, Colin M, Buee L, Panayi F, Diguet E, Brouillet E, Hantraye P, Bemelmans AP, Cambon K (2018). Potentiating tangle formation reduces acute toxicity of soluble tau species in the rat. Brain.

[6] Dejanovic B, Huntley MA, De Maziere A, Meilandt WJ, Wu T, Srinivasan K, Jiang ZY, Gandham V, Friedman BA, Ngu H, Foreman O, Carano RAD, Chih B, Klumperman J, Bakalarski C, Hanson JE, Sheng M (2018). Changes in the synaptic proteome in tauopathy and rescue of Tau-induced synapse loss by C1q antibodies. Neuron.

[7] du Sert NP, Hurst V, Ahluwalia A, Alam S, Avey MT, Baker M, Browne WJ, Clark A, Cuthill IC, Dirnagl U, Emerson M, Garner P, Holgate ST, Howells DW, Karp NA, Lazic SE, Lidster K, MacCallum CJ, Macleod M, Pearl EJ, Petersen OH, Rawle F, Reynolds P, Rooney K, Sena ES, Silberberg SD, Steckler T, Wurbel H (2020). The ARRIVE guidelines 2.0: updated guidelines for reporting animal research. BMC Vet Res.

[8] Garcia-Alloza M, Robbins EM, Zhang-Nunes SX, Purcell SM, Betensky RA, Raju S, Prada C, Greenberg SM, Bacskai BJ, Frosch MP (2006). Characterization of amyloid deposition in the APPswe/PS1dE9 mouse model of Alzheimer disease. Neurobiol Dis.

[9] Gary C, Lam S, Herard AS, Koch JE, Petit F, Gipchtein P, Sawiak SJ, Caillierez R, Eddarkaoui S, Colin M, Aujard F, Deslys JP, Network FN, Brouillet E, Buée L, Comoy EE, Pifferi F, Picq J-L, Dhenain M (2019). Encephalopathy induced by Alzheimer brain inoculation in a non-human primate. Acta Neuropathol Commun.

[10] Gilles JF, Dos Santos M, Boudier T, Bolte S, Heck N (2017). DiAna, an ImageJ tool for object-based 3D co-localization and distance analysis. Methods.

[11] He ZH, Guo JL, McBride JD, Narasimhan S, Kim H, Changolkar L, Zhang B, Gathagan RJ, Yue CY, Dengler C, Stieber A, Nitla M, Coulter DA, Abel T, Brunden KR, Trojanowski JQ, Lee VMY (2018). Amyloid-beta plaques enhance Alzheimer's brain tau-seeded pathologies by facilitating neuritic plaque tau aggregation. Nat Med.

[12] Hoover BR, Reed MN, Su J, Penrod RD, Kotilinek LA, Grant MK, Pitstick R, Carlson GA, Lanier LM, Yuan LL, Ashe KH, Liao D (2010). Tau mislocalization to dendritic spines mediates synaptic dysfunction independently of neurodegeneration. Neuron.

[13] Kane MD, Lipinski WJ, Callahan MJ, Bian F, Durham RA, Schwarz RD, Roher AE, Walker LC (2000). Evidence for seeding of beta-amyloid by intracerebral infusion of Alzheimer brain extracts in beta-amyloid precursor protein-transgenic mice. J Neurosci.

[14] Lam S, Petit F, Hérard A-S, Boluda S, Eddarkaoui S, Guillermier M, Network T-C, Buée L, Duyckaerts C, Haïk S, Picq J-L, Dhenain M (2021). Transmission of amyloid-beta and tau pathologies is associated with cognitive impairments in a primate. Acta Neuropathol Commun.

[15] Leyns CEG, Gratuze M, Narasimhan S, Jain N, Koscal LJ, Jiang H, Manis M, Colonna M, Lee VMY, Ulrich JD, Holtzman DM (2019). TREM2 function impedes tau seeding in neuritic plaques. Nat Neurosci.

[16] Metaxas A, Thygesen C, Kempf SJ, Anzalone M, Vaitheeswaran R, Petersen S, Landau AM, Audrain H, Teeling JL, Darvesh S, Brooks DJ, Larsen MR, Finsen B (2019). Ageing and amyloidosis underlie the molecular and pathological alterations of tau in a mouse model of familial Alzheimer's disease. Sci Rep.

[17] Meyer-Luehmann M, Coomaraswamy J, Bolmont T, Kaeser S, Schaefer C, Kilger E, Neuenschwander A, Abramowski D, Frey P, Jaton AL, Vigouret JM, Paganetti P, Walsh DM, Mathews PM, Ghiso J, Staufenbiel M, Walker LC, Jucker M (2006). Exogenous induction of cerebral beta-amyloidogenesis is governed by agent and host. Science.

[18] Nelson PT, Alafuzoff I, Bigio EH, Bouras C, Braak H, Cairns NJ, Castellani RJ, Crain BJ, Davies P, Del Tredici K, Duyckaerts C, Frosch MP, Haroutunian V, Hof PR, Hulette CM, Hyman BT, Iwatsubo T, Jellinger KA, Jicha GA, Kovari E, Kukull WA, Leverenz JB, Love S, Mackenzie IR, Mann DM, Masliah E, McKee AC, Montine TJ, Morris JC, Schneider JA, Sonnen JA, Thal DR, Trojanowski JQ, Troncoso JC, Wisniewski T, Woltjer RL, Beach TG (2012). Correlation of Alzheimer disease neuropathologic changes with cognitive status: a review of the literature. J Neuropath Exp Neur.

[19] Noda-Saita K, Terai K, Iwai A, Tsukamoto M, Shitaka Y, Kawabata S, Okada M, Yamaguchi T (2004). Exclusive association and simultaneous appearance of congophilic plaques and AT8-positive dystrophic neurites in Tg2576 mice suggest a mechanism of senile plaque formation and progression of neuritic dystrophy in Alzheimer's disease. Acta Neuropathol.

[20] Paxinos G, Franklin KBJ (2001). The mouse brain in stereotaxic coordinates.

[21] Pooler AM, Noble W, Hanger DP (2014). A role for tau at the synapse in Alzheimer's disease pathogenesis. Neuropharmacology.

[22] Radde R, Bolmont T, Kaeser SA, Coomaraswamy J, Lindau D, Stoltze L, Calhoun ME, Jaggi F, Wolburg H, Gengler S, Haass C, Ghetti B, Czech C, Holscher C, Mathews PM, Jucker M (2006). Abeta42-driven cerebral amyloidosis in transgenic mice reveals early and robust pathology. EMBO Rep.

[23] Roberson ED, Scearce-Levie K, Palop JJ, Yan F, Cheng IH, Wu T, Gerstein H, Yu GQ, Mucke L (2007). Reducing endogenous tau ameliorates amyloid beta-induced deficits in an Alzheimer's disease mouse model. Science.

[24] Sanchez-Mejias E, Navarro V, Jimenez S, Sanchez-Mico M, Sanchez-Varo R, Nunez-Diaz C, Trujillo-Estrada L, Davila JC, Vizuete M, Gutierrez A, Vitorica J (2016). Soluble phospho-tau from Alzheimer's disease hippocampus drives microglial degeneration. Acta Neuropathol.

[25] Selkoe DJ (2002). Alzheimer's disease is a synaptic failure. Science.

[26] Sivanesan S, Tan A, Rajadas J (2013). Pathogenesis of abeta oligomers in synaptic failure. Curr Alzh Res.

[27] Spires-Jones TL, Hyman BT (2014). The intersection of amyloid beta and tau at synapses in Alzheimer's disease. Neuron.

[28] Streit WJ, Braak H, Xue QS, Bechmann I (2009). Dystrophic (senescent) rather than activated microglial cells are associated with tau pathology and likely precede neurodegeneration in Alzheimer's disease. Acta Neuropathol.

[29] Terry RD, Masliah E, Salmon DP, Butters N, DeTeresa R, Hill R, Hansen LA, Katzman R (1991). Physical basis of cognitive alterations in Alzheimer's disease: synapse loss is the major correlate of cognitive impairment. Ann Neurol.

[30] Vergara C, Houben S, Suain V, Yilmaz Z, De Decker R, Vanden Dries V, Boom A, Mansour S, Leroy K, Ando K, Brion JP (2019). Amyloid-pathology enhances pathological fibrillary tau seeding induced by Alzheimer PHF in vivo. Acta Neuropathol.

[31] Ye L, Hamaguchi T, Fritschi SK, Eisele YS, Obermuller U, Jucker M, Walker LC (2015). Progression of seed-induced Abeta deposition within the limbic connectome. Brain Pathol.

