# Supplementary material for: Pathological changes induced by Alzheimer’s brain inoculation in amyloid-beta plaque-bearing mice
Source: Acta Neuropathol Commun. 2022 Aug 16;10:112. doi: 10.1186/s40478-022-01410-y (PMC9380345; doi:10.1186/s40478-022-01410-y)
Supplement: Supplementary file 1 — Additional file 1. The additional file contains Table S1 and Figures S1-S9. [file 40478_2022_1410_MOESM1_ESM.docx]

# **Pathological changes induced by Alzheimer’s brain inoculation in amyloid-beta plaque-bearing mice**

# **Supplementary data**

Suzanne Lam, Anne-Sophie Hérard, Susana Boluda, Fanny Petit, Sabiha Eddarkaoui, Karine Cambon, The Brainbank Neuro-CEB Neuropathology Network, Jean-Luc Picq, Luc Buée, Charles Duyckaerts, Stéphane Haïk, Marc Dhenain

**Supplementary Table 1. Patient characteristics**

| 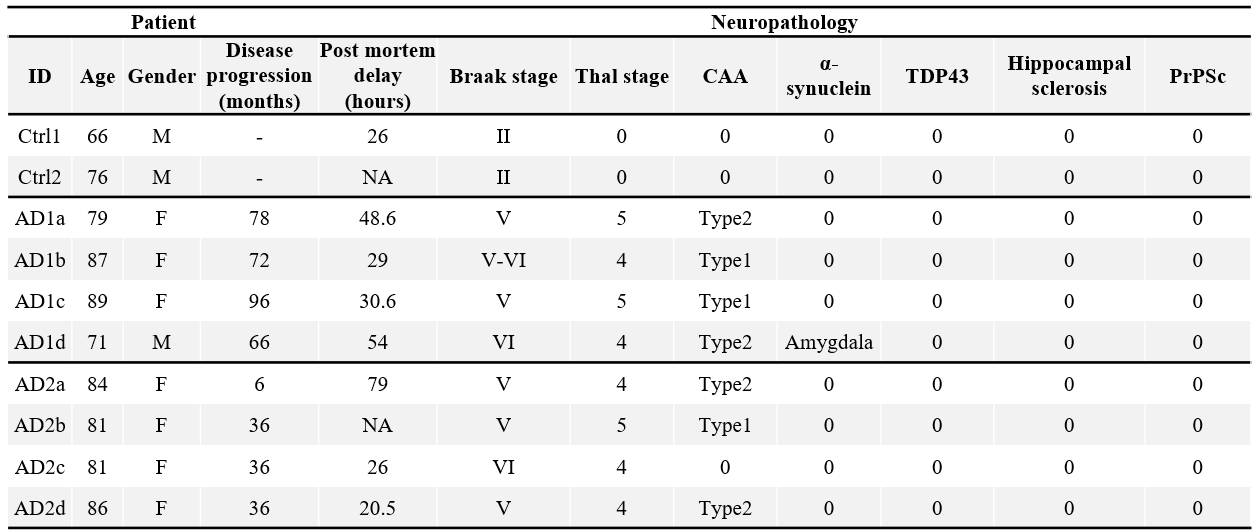 |
| --- |
| Age-matched classical slowly (AD1) and rapidly evolving (AD2) Alzheimer patients were selected based on disease duration (over or under 36 months) and neuropathological evaluation, including similar Braak and Thal stages. Brains were negative for α-synuclein, TAR DNA-binding protein 43 (TDP43), hippocampal sclerosis and pathological prion PrPSc. Two non-Alzheimer control individuals (Ctrl) were also included in this study. NA: not available. |

| \| 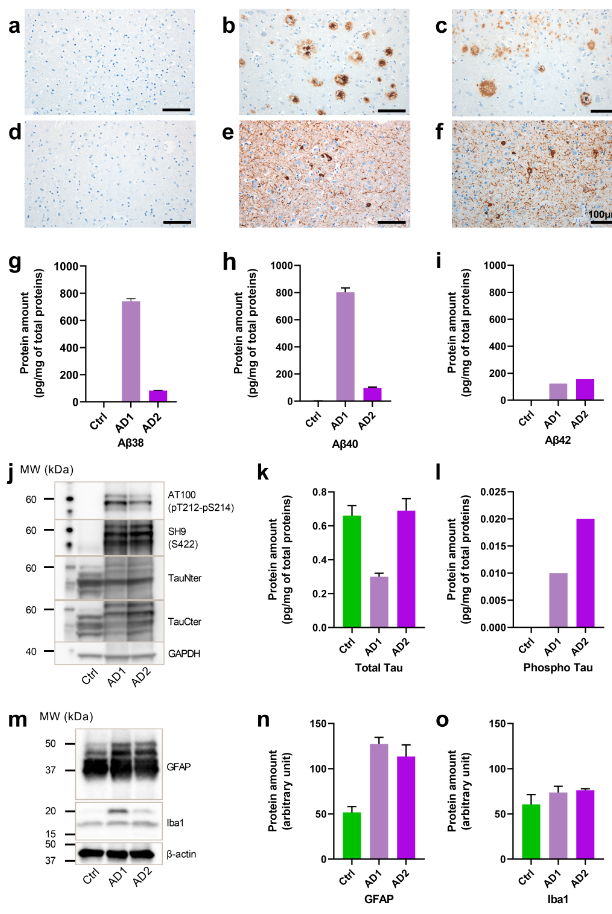 \| \| --- \| \| **Supplementary Figure 1. Characteristics of human brain samples and brain extracts inoculated to animals.** Representative images of Ctrl, AD1 and AD2 brain samples stained for Aβ (**a-c**) and tau (**d-f**) pathologies. Scale bars = 100 µm. Three brain extracts were prepared from 2 control individuals, 4 cases with a slowly evolving form of AD and 4 cases with a rapidly evolving form of AD (Ctrl, AD1 and AD2 brain extracts, respectively). All quantifications of the brain extracts (Ctrl, AD1 or AD2) were performed in duplicate and data are shown as mean ± standard deviation of the replicates. (**g-i**) Quantifications of total Aβ38, Aβ40 and Aβ42 of the brain extracts (MSD technology). Both AD brain extracts had more Aβ proteins compared to the Ctrl one. The AD1 extract showed more Aβ38 and Aβ40 than the AD2 one. (**j-l**) Tau profile evaluation by western blot revealed a pathological hyperphosphorylated tau triplet at 60, 64 and 69 kDa observed in AD and a typical shift in the molecular weight of the Alzheimer Tau-Cter triplet in AD1 and AD2 brain extracts (**j**). Total tau (**k**) and pathological phospho-tau 181 levels (**l**) were assessed using ELISA quantification. Neuroinflammatory profile evaluation by western blots revealed higher astrocytic presence (GFAP-positive) in AD1 and AD2 brain extracts compared to the Ctrl extract (**m-n**). Microglial (Iba1-positive) levels were similar in the Ctrl, AD1 and AD2 groups (**m, o**). (data already published in (Lam, Petit et al, Acta Neuropathol Com, 2021)). \| |
| --- | --- | --- |

| 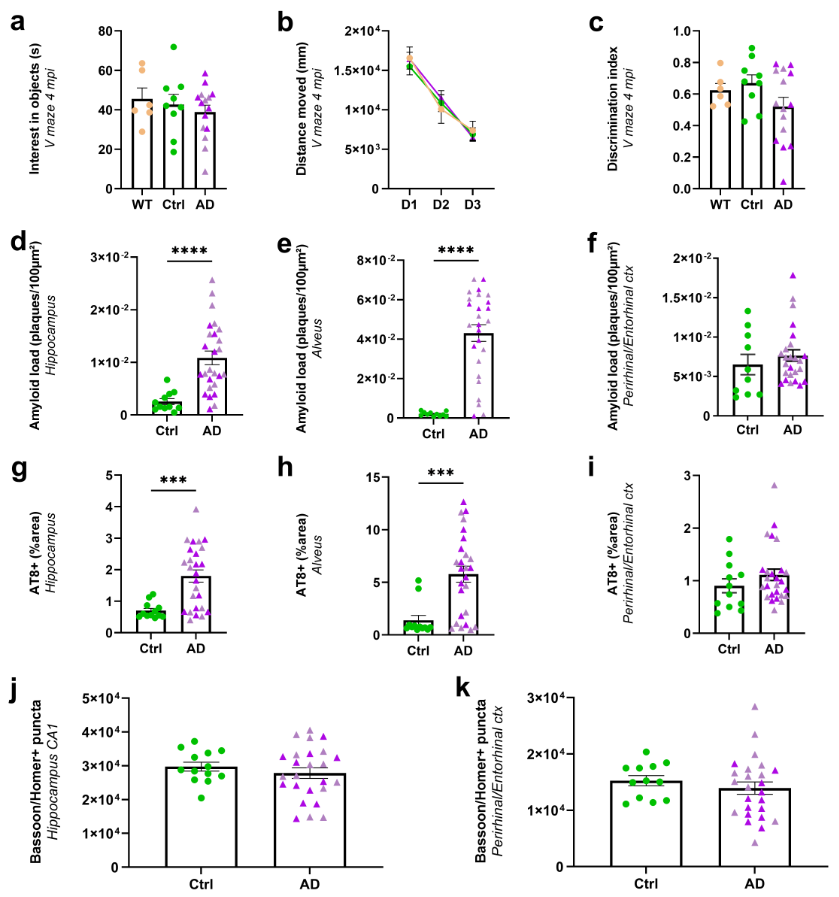 |
| --- |
| **Supplementary Figure 2. Cognitive performances, Aβ and tau loads, and synaptic density following AD_be_ inoculation in APP_swe_/PS1_dE9_ mice (4 mpi).** (**a-c**) Object recognition performances were evaluated at 4 mpi using a V-maze test. WT mice and APP_swe_/PS1_dE9_ mice inoculated with Ctrl_be_ or AD_be_ had comparable exploratory activity, as suggested by the time spent on exploring the objects (**a**) (*p*>0.05; Kruskal-Wallis with Dunn’s multiple comparisons) and the distance moved throughout the 3-day test (**b**) (for the days: *F_(1.9, 51.7)_*=131.2, *p*<0.0001; for the groups: *F_(2, 27)_*=0.06, *p*=0.9; two-way repeated measures ANOVA with the Geisser-Greenhouse correction and Dunnett’s multiple comparisons). No difference in the novel object recognition test was reported between the groups, as similar discrimination indexes were observed (**c**) (*p*>0.05; Kruskal-Wallis with Dunn’s multiple comparisons). (**d-f**) Aβ load quantification at 4 mpi revealed that AD_be_ inoculation accelerates Aβ deposition in the hippocampus (**d**, *p*<0.0001; Mann Whitney's test) and alveus (**e**, *p*<0.0001), but not the perirhinal/entorhinal cortex (**f**, *p*=0.3). (**g-i**) AT8-positive tau overall quantification at 4 mpi revealed that AD_be_ inoculation induces tau lesions in the hippocampus (**g,** *p*= 0.0007), in the alveus (**h**, *p*= 0.0003) but not the perirhinal/entorhinal cortex (**i**, *p*=0.2). (**j-k**) Quantification of Bassoon and Homer colocalization at 4 mpi did not show any differences in the CA1 (**j**) and in the perirhinal/entorhinal cortex (**k**) between the groups (*p*>0.05; Mann Whitney's test). ****p*<0.001; *****p*<0.0001. *n_Ctrl_*=9, *n_AD1_*=7 (light pink), *n_AD2_*=8 (dark pink), *n_WT_*=6 mice in a-c, *n_Ctrl_*=11, *n_AD1_*=14 (light pink), *n_AD2_*=12 (dark pink) in d-k. Data are shown as mean ± s.e.m. |


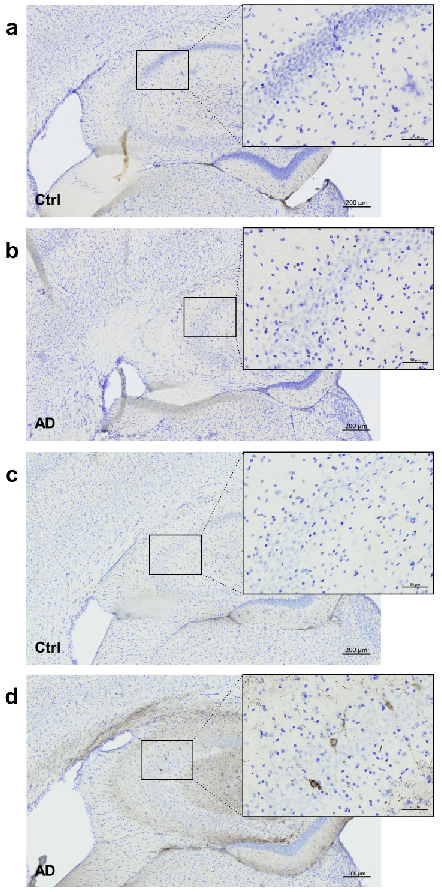


**Supplementary Figure 3. AT8 immunolabelling in the hippocampus of Ctrl_be_- (a, c) and AD_be_-inoculated (b, d) APP_swe_/PS1_dE9_ mice** **at 1 mpi (a-b) and 8 mpi (c-d).** AT8-positive tau lesions were not detected in 1 mpi APP_swe_/PS1_dE9_ mice (b) while they were detected at 8mpi (d). Scale bars = 200 µm (**a-d**) and 50 µm (**a-d insets**).

| 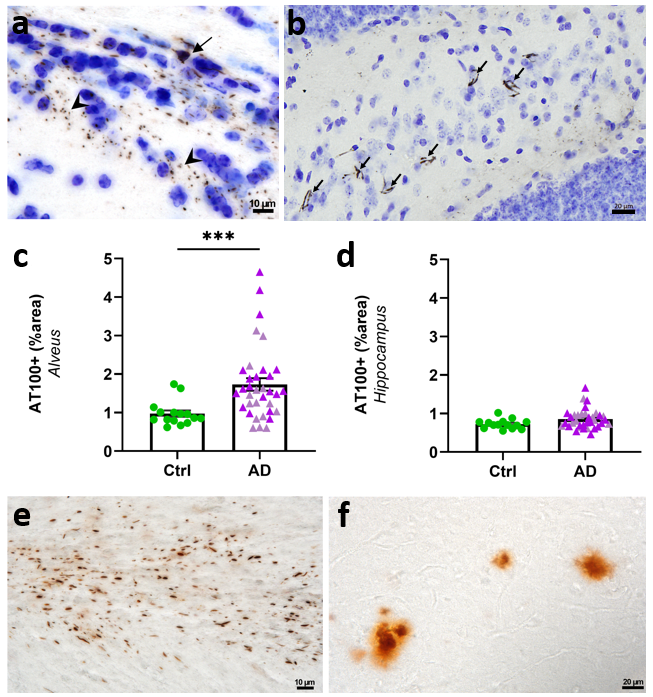 |
| --- |
|  |
| **Supplementary Figure 4. AT100 and Gallyas-positive lesions next to the inoculation site of APP_swe_/PS1_dE9_ mice 8 months after AD_be_ infusion.** (**a-b**) AT100 staining revealed neuropil threads (arrowheads) as well as labelling of cell bodies (arrows). (**c-d**) Overall quantification of AT100 labelling showed increased labelling in the alveus (**c**) but not in the rest of the hippocampus (**d**) of AD_be_-inoculated mice (*p*=0.0007 and 0.092 respectively, Mann-Whitney test). Gallyas silver staining revealed neuropil threads (**e**) as well as Aβ plaques (**f**) in AD_be_-inoculated mice. *n_Ctrl_*=15, *n_AD1_*=15 (light pink), *n_AD2_*=20 (dark pink). Data are shown as mean ± s.e.m. Scale bars = 10 µm (**a, e**) and 20 µm (**b, f**). |

| 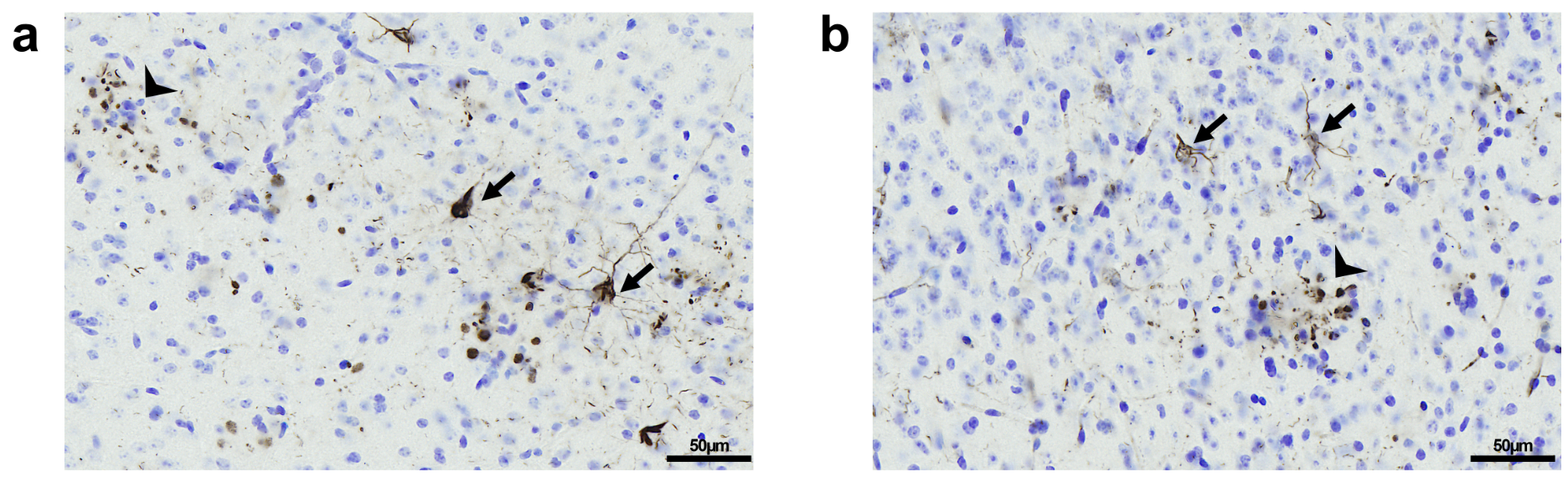 |
| --- |
| **Supplementary Figure 5. AT8-positive lesions in the visual cortex of APP_swe_/PS1_dE9_ mice 8 months after AD_be_ infusion.** AT8 staining revealed NFTs (arrows) surrounded by neuropil threads, as well as neuritic plaques (arrowheads), in the visual cortex of AD brain-inoculated mice (**a**, **b**). Scale bars = 50 µm. |

| 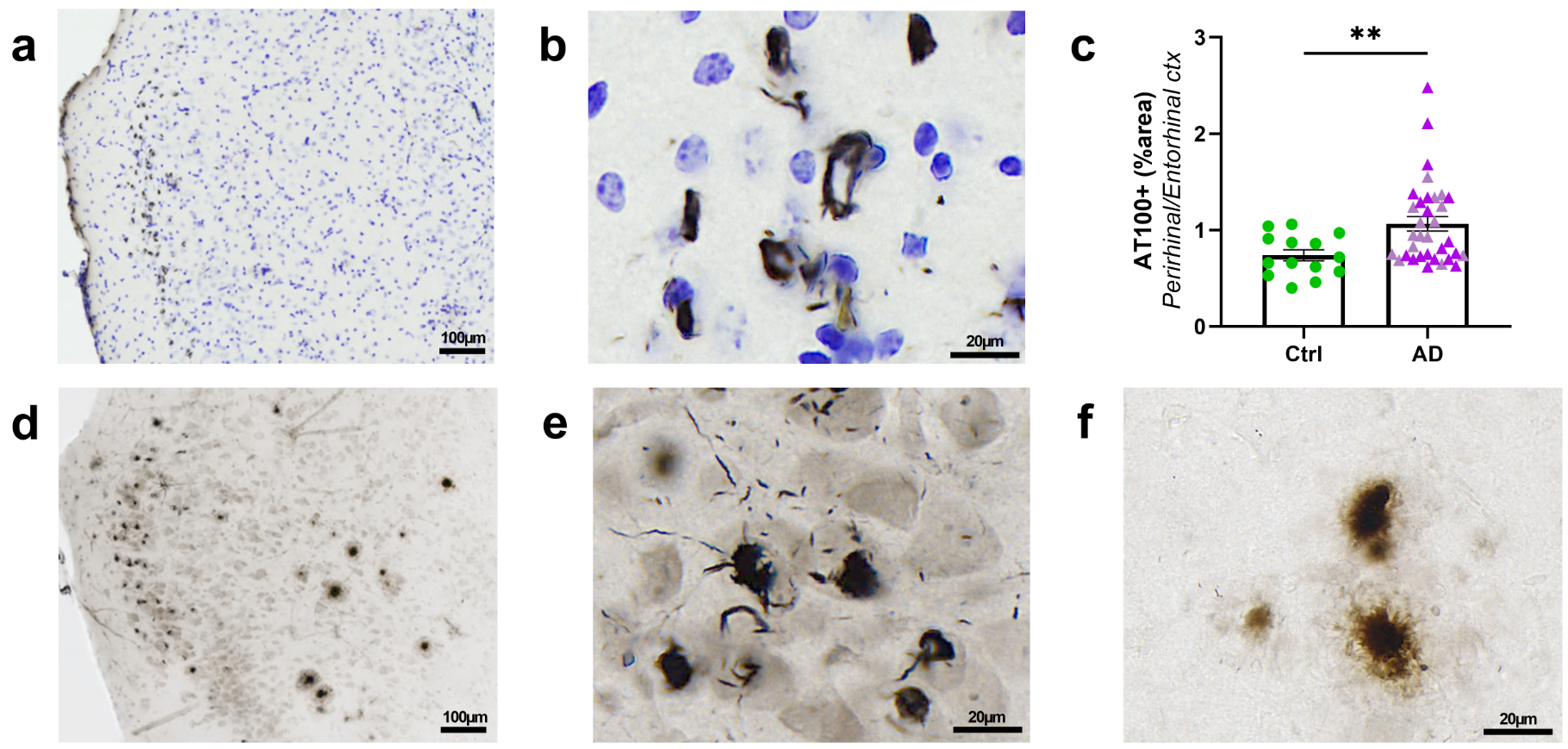 |
| --- |
| **Supplementary Figure 6. AT100- and Gallyas-positive lesions spreading to the perirhinal/entorhinal cortex of APP_swe_/PS1_dE9_ mice 8 months after AD_be_ infusion.** (**a-b**) AT100 staining revealed NFTs in the perirhinal/entorhinal cortex. (**c**) Overall quantification of AT100 labelling showed increased labelling in the perirhinal/entorhinal cortex of AD_be_-inoculated mice compared to Ctrl_be_-inoculated ones (*p*=0.005, Mann-Whitney test). (**d-f**) Gallyas silver staining revealed labelled neurons evoking NFTs (**e**) as well as Aβ plaques (**f**). *n_Ctrl_*=15, *n_AD1_*=15 (light pink), *n_AD2_*=20 (dark pink). Data are shown as mean ± s.e.m. Scale bars = 100 µm (**a, d**) and 20 µm (**b, e, f**). |


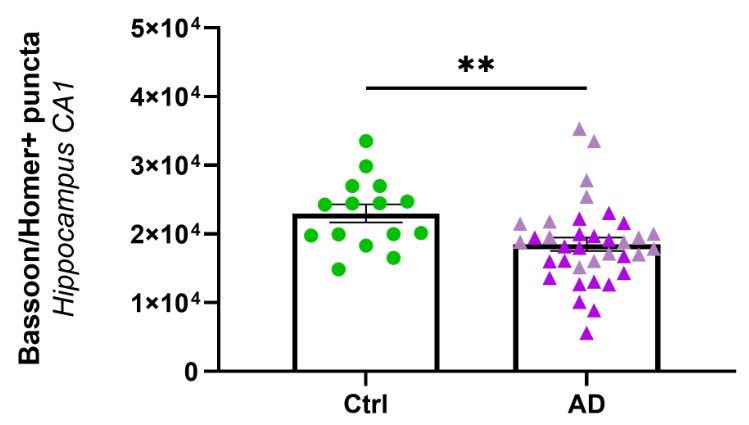


**Supplementary Figure 7. Synaptic density is decreased in the CA1 of AD_be_-inoculated APP_swe_/PS1_dE9_ mice compared to Ctrl_be_-inoculated mice at 8 mpi (Mann Whitney's test, p=0.005).** *n_Ctrl_*=15, *n_AD1_*=15 (light pink), *n_AD2_*=20 mice (dark pink). ***p*<0.01; Data are shown as mean ± s.e.m.

| 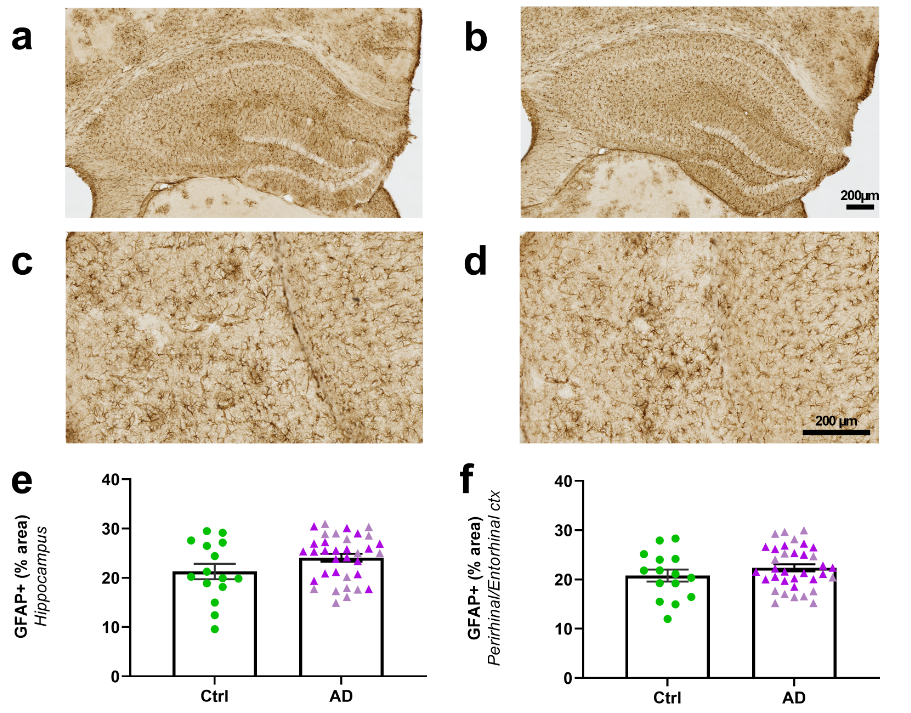 |
| --- |
| **Supplementary Figure 8. Astrocytic coverage was similar in the hippocampus and perirhinal/entorhinal cortex of AD_be_- and Ctrl_be_-inoculated APP_swe_/PS1_dE9_ mice at 8 mpi.** GFAP staining revealed similar astrocytic loads in the hippocampus (**a-b, e**) and perirhinal/entorhinal cortex (**c-d, f**) of AD_be_- and Ctrl_be_-inoculated APP_swe_/PS1_dE9_ mice at 8 mpi. *n_Ctrl_*=15, *n_AD1_*=15 (light pink), *n_AD2_*=20 mice (dark pink). Data are shown as mean ± s.e.m. Scale bars = 200 µm. |


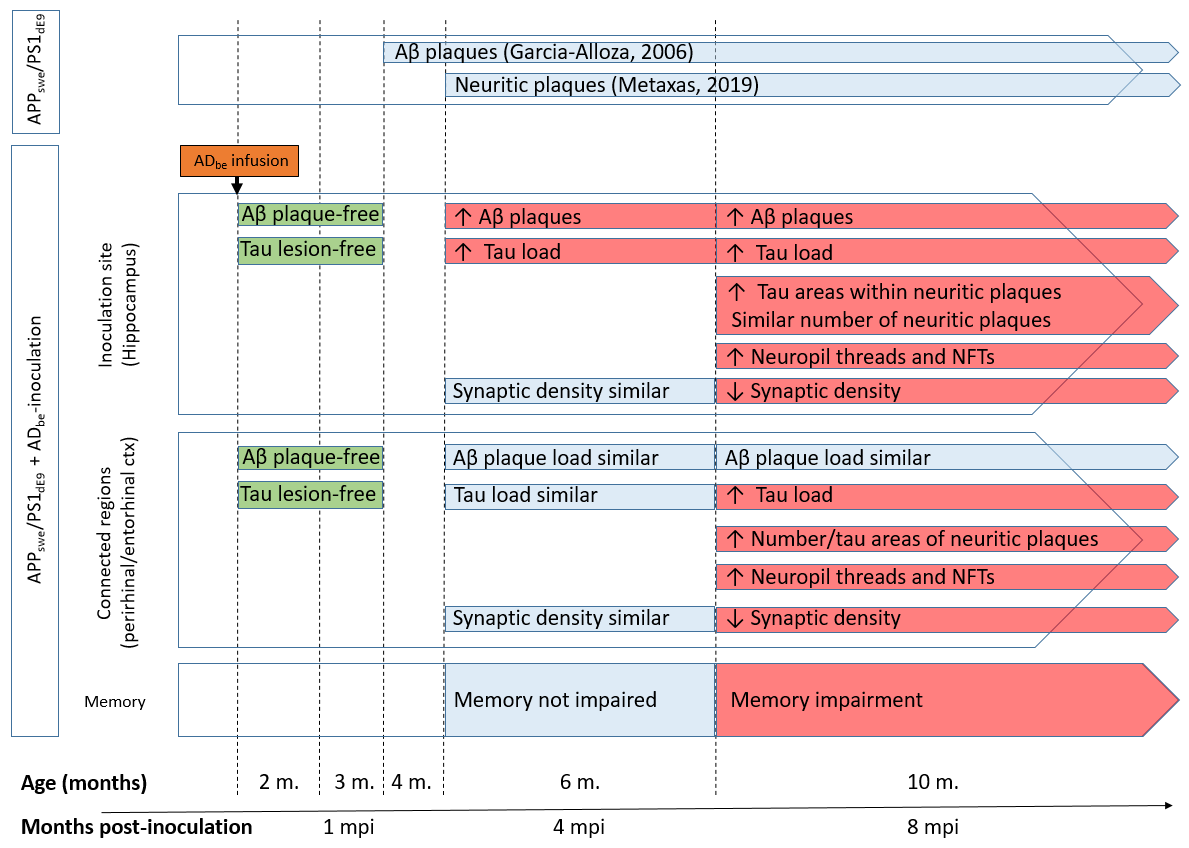


**Supplementary Figure 9. Overview of the changes reported after infusion of AD_be_ in APP_swe_/PS1_dE9_.** The first lines (APP_swe_/PS1_dE9_) display published timeframes for occurrence of Aβ plaques (4 months of age) and tau-positive neuritic plaques (6 months of age) in APP_swe_/PS1_dE9_ mice. The lines below (APP_swe_/PS1_dE9_+AD_be_) display events reported in AD_be_-inoculated APP_swe_/PS1_dE9_ mice compared to Ctrl_be_-inoculated APP_swe_/PS1_dE9_ animals. One month post inoculation (mpi), Aβ and tau were not detected in AD_be_-inoculated APP_swe_/PS1_dE9_ mice. Blue frames display events that were similar in AD_be_- and Ctrl_be_-inoculated APP_swe_/PS1_dE9_ animals. Red frames highlight impairments detected in AD_be_-inoculated APP_swe_/PS1_dE9_ mice. First changes concerned increased Aβ and tau load within the hippocampus at 4 mpi. Synaptic density and memory were not modified at this stage. Then at 8 mpi, increased Aβ and tau load continued to involve the hippocampus and tau pathology extended to connected regions (perirhinal/entorhinal cortex). Synaptic density was reduced in the hippocampus and perirhinal/entorhinal cortex and memory was impaired at this stage.
